# Permanences d’accès aux soins de santé (PASS): dispositifs de prise en charge des plus précaires. Exemple de la PASS de l’Hôtel-Dieu de Paris

**DOI:** 10.48327/mtsi.v5i4.2025.729

**Published:** 2025-08-06

**Authors:** Alexandra REHBINDER, Hélène de CHAMPS-LÉGER, Louis CROZIER, Guillaume RIEUTORD, Hélène LELONG

**Affiliations:** Hôpital Hôtel Dieu, Centre de diagnostic et de thérapeutique, Permanence d’accès aux Soins de Santé, 1, place du Parvis Notre Dame, 75004 Paris, France

**Keywords:** PASS, Aide médicale de l’État (AME), Hôtel-Dieu, Assistance publique - Hôpitaux de Paris (APHP), Précarité, Vulnérabilité, Exclusion, Sans-abris, Migrants, Accès aux soins, Couverture sociale, Paris, France, Europe, PASS, State medical aid (AME), Hôtel-Dieu, Assistance publique -Hôpitaux de Paris (APHP), Precariousness, Vulnerability, Exclusion, Homelessness, Migrants, Access to healthcare, Social security coverage, Paris, France, Europe

## Abstract

Les PASS (Permanences d’accès aux soins de santé) ont été créées en France par la loi de 1998 relative à la lutte contre les exclusions, afin de garantir l’accès aux soins pour tous, incluant les personnes en situation de précarité. Elles visent à répondre aux multiples freins, sociaux, économiques, administratifs et linguistiques, qui éloignent du système de santé ces populations en situation de vulnérabilité.

L’article présente ici une synthèse du fonctionnement des PASS en France et détaille l’exemple de la PASS de l’Hôtel-Dieu (Assistance publique - Hôpitaux de Paris APHP), qui offre aux patients une approche holistique, incluant soins médicaux, suivi psychiatrique, accompagnement social, interprétariat, et consultations spécialisées. En 2023, la France comptait 451 PASS. La PASS de l’Hôtel-Dieu, forte d’une équipe pluridisciplinaire, a réalisé plus de 10 500 consultations médicales pour 4 144 patients différents. Les usagers sont majoritairement des hommes jeunes, en situation de migration, sans couverture maladie (69%) ni hébergement stable (près de 90%). Les pathologies les plus fréquentes incluent des maladies chroniques (HTA, diabète), infectieuses (hépatites B/C, VIH, tuberculose) et psychiques (stress post-traumatique, anxiété).

Lexemple de l’Hôtel-Dieu illustre les spécificités des PASS: accessibilité sans rendez-vous, unité de lieu et de temps, adaptation aux temporalités précaires. Ces dispositifs permettent de rétablir un lien avec le système de soins pour des populations très vulnérables. Leur efficacité dépend d’un financement pérenne, d’une coordination médico-sociale renforcée et d’une reconnaissance institutionnelle de leur rôle.

Les PASS répondent à un impératif éthique et de santé publique: soigner les plus éloignés du système de soins. Leur pérennité nécessite des moyens humains et financiers adaptés. Elles doivent rester un lieu d’innovation, d’accueil et d’inclusion, s’adaptant en permanence aux mutations sociales, politiques et migratoires.

## Abstract (en)

The 1998 French law on combating exclusion established PASS units (Permanences d’Accès aux Soins de Santé, or Healthcare Access Points) to guarantee access to healthcare for all, including people in precarious situations. PASS centers aim to address the social, economic, administrative, and linguistic barriers that prevent vulnerable populations from accessing the healthcare system.

This article provides an overview of how PASS operate in France and describes the PASS at Hôtel-Dieu in Paris (Public Hospitals of Paris). This PASS offers patients a holistic approach that includes medical care, psychiatric follow-up, social support, interpretation services, and specialized consultations.

As of 2023, there were 451 PASS centers in France. The Hôtel-Dieu PASS, with its multidisciplinary team, carried out over 10,500 medical consultations for 4,144 patients. Most users are young male migrants without health insurance (69%) or stable housing (nearly 90%). The most common conditions are chronic diseases (e.g., hypertension and diabetes), infectious diseases (e.g., hepatitis B/C, HIV, and tuberculosis), and mental health issues (e.g., post-traumatic stress disorder and anxiety).

The Hôtel-Dieu example illustrates the specific features of PASS centers: accessibility without appointments, single locations and time slots, and adaptability to precarious schedules. These services enable vulnerable populations to reconnect with the healthcare system. However, their effectiveness depends on long-term funding, enhanced medical and social coordination, and institutional recognition of their role.

PASS units respond to an ethical and public health imperative: caring for those furthest from the healthcare system. Their sustainability requires adequate human and financial resources. They must remain places of innovation, welcome, and inclusion that constantly adapt to social, political, and migratory changes.

## Introduction

*Precarius* signifie étymologiquement « mal assuré » et plus largement « est précaire » ce qui est fragile, incertain. Être en situation de précarité, c’est vivre exposé à une incertitude permanente, à une instabilité dans le temps et l’espace, sans aucune assurance. Plusieurs domaines y contribuent dont l’absence de logement, d’activité professionnelle, de couverture maladie, de ressources financières. Être malade et en situation de précarité est une double peine qui complexifie la prise en charge médicale et fragilise les parcours de soins. Fortes de ces constats et face à l’accroissement du taux de pauvreté (16% en 1996) [5], les autorités publiques françaises se sont saisies du sujet: la lutte contre les exclusions, par la loi du 29 juillet 1998 devient un impératif national fondé sur le respect de l’égale dignité de tous les êtres humains [20].

Cette loi « tend à garantir, sur l’ensemble du territoire, l’accès effectif de tous aux droits fondamentaux », dont celui de la protection de la santé. Elle promeut la création de dispositifs médicaux spécifiquement dédiés aux plus démunis (précaires) dont ceux sans couverture sociale (certains étrangers en séjour irrégulier par exemple): les Permanences d’accès aux soins de santé, dites PASS, une spécificité française, dont fait partie la PASS de l’Hôtel-Dieu (Assistance publique - Hôpitaux de Paris).

Afin d’assurer cet « accès effectif de tous », ces dispositifs s’adaptent aux contraintes et aux difficultés propres des personnes en situation de précarité et visent ainsi à faciliter l’accès au système de santé et à accompagner les démarches nécessaires à la reconnaissance des droits des plus démunis. Au moins deux activités sont constitutives d’une PASS, les soins immédiats et l’accès à une protection maladie à moyen terme. Récemment, la menace de suppression ou de limitation de l’aide médicale de l’État (AME), suite à la loi « immigration, intégration, asile » du 26 janvier 2024, a remis en question l’accès effectif de tous aux soins de santé. Si l’AME n’a pas été supprimée, la loi de finances pour 2025, votée au Sénat, restreint néanmoins l’enveloppe dédiée à l’AME de 111 millions d’euros par rapport au projet initial [8,21].

L’article présente en premier lieu l’organisation des PASS en France, puis s’attache à détailler l’exemple de la PASS de l’Hôtel-Dieu. Dans la discussion sont abordés la spécificité et les grands principes de la prise en charge des plus vulnérables.

## Les PASS en France, 25 ans d’existence

En 2023, on dénombrait 451 PASS réparties sur le territoire national. Elles obéissent à la note d’instruction et au cahier des charges de la direction générale de l’offre de soins (DGOS) [10,12].

### Les différents types de PASS

La grande majorité d’entre elles (380) sont généralistes. On en distingue 4 types.

- Les « PASS dédiées » proposent un accueil par des soignants spécifiques dans un lieu centralisé bien identifié. Elles sont composées *a minima* du trinôme assistant social/ infirmier/médecin. Les dispositifs centralisés assurent une prise en charge globale par l’accès à une consultation médicale généraliste, à un plateau technique, aux soins infirmiers, aux médicaments et aux consultations de spécialité. Des activités complémentaires (équipes mobiles, soins bucco-dentaires, prise en charge mère/enfant…), réalisées par du personnel dédié, peuvent s’y ajouter. Ces PASS peuvent consolider l’offre de soins proposée en ville ou se déployer en milieu hospitalier. Elles ont pour vocation de faire le lien entre l’hôpital et la ville pour l’accompagnement médico-social global des personnes en situation de précarité dans la perspective d’un retour à des consultations classiques.
- Les « PASS transversales » interviennent sur l’ensemble de l’hôpital, notamment après une consultation aux urgences qui est souvent le lieu de premier recours des patients en situation de précarité. Elles correspondent aux consultations réalisées au sein de chaque service, pour des patients présentant une situation médico-sociale complexe. La circulaire du 12 avril 2022 relative au cahier des charges des PASS prévoit leur disparition progressive au motif que cette activité relève du budget des Fonds d’investissement régional précarité [12].
- Les « PASS mobiles » interviennent « hors les murs ». Elles agissent au plus près des patients vulnérables, en allant proactivement vers les personnes les plus éloignées du système de santé: dans les centres d’accueil et d’accompagnement à la réduction des risques pour usagers de drogues, en centres d’accueil pour demandeurs d’asile, en campements. Des « bus santé » sont financés dans cette optique. Cette démarche d’« aller-vers » a pour objectif d’identifier les publics en situation de vulnérabilité, de faire émerger leur demande de prise en charge médicale et de les orienter vers les structures médicales les mieux adaptées à leur situation.
- Enfin, il existe des PASS spécialisées dont 2 en ophtalmologie, 10 en soins bucco-dentaires, et 44 en milieu psychiatrique.

### Création des PASS de ville

Les PASS de ville ont vu le jour en 2022 après l’expérimentation fructueuse de la création en 2013 des PASS de villes franciliennes, à l’initiative du programme régional d’accès à la prévention et aux soins. Elles ont été pensées pour étayer le maillage territorial de l’accès aux soins de premier recours en médecine de ville des personnes les plus vulnérables.

De par leur ancrage territorial et leur approche de réseau envers les acteurs de proximité, les PASS de ville sont venues compléter l’offre de soins des PASS hospitalières.

On dénombre à ce jour 18 opérateurs en Île-de-France selon deux modalités de déploiements: en centres et maisons de santé, et *via* les associations porteuses des ex-réseaux de santé précarité. Ces PASS de ville facilitent l’accès aux soins en proposant un accès non seulement aux consultations médicales, mais également aux consultations infirmières et à la délivrance des médicaments, rendues possibles grâce à la coopération avec les Caisses primaires d’assurance maladie (CPAM) du territoire. Le volet social n’est pas négligé; un entretien préalable avec un(e) travailleur(se) social(e) permet le repérage des vulnérabilités, l’accompagnement social dans les démarches d’ouverture de droits et la gestion des démarches de recouvrement des frais liés aux actes de soins délivrés.

La création des PASS de ville offre l’avantage majeur d’une offre de proximité, proche du lieu d’ancrage des patients, facilitant la continuité des soins surtout après l’ouverture des droits.

**Figure 1:**
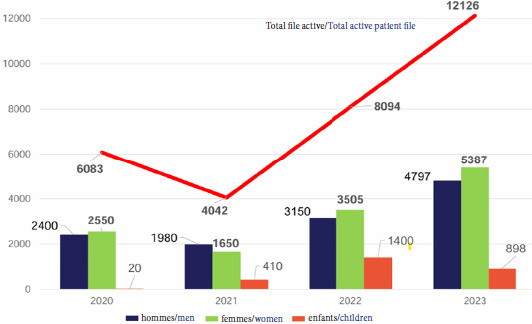
File active recensée pour les hommes, les femmes et les enfants des PASS de ville en Île-de-France de 2020 à 2023

Source: restitution de la DGOS de la Journée Régionale des PASS 2025

Les Agences régionales de santé (ARS) financent ces dispositifs à hauteur de 1 390 000 €. L’évolution croissante des files actives (49% d’augmentation en 2023 par rapport à 2020) met en avant le besoin croissant et l’efficacité de ces structures (Fig. 1) [1].

### Public ciblé

Les PASS sont destinées à toute personne en situation de précarité nécessitant une prise en charge médicale et dont la situation psycho-médico-sociale freine leur intégration au système de soins [12]:

- en raison d’une couverture sociale inexistante ou insuffisante, rendant impossible la prise en charge des frais médicaux;
- pour d’autres motifs d’ordre social (difficulté à recourir spontanément au système de santé, besoin d’accompagnement dans le parcours de soins, situation de marginalisation ou de forte désocialisation, barrière majeure de la langue, inquiétudes quant au statut juridique d’irrégularité sur le territoire ou encore consultations complexes liées à des troubles psychiques ou psychiatriques).

À travers l’accompagnement global des patients, la PASS incarne un dispositif passerelle, qui offre un accompagnement temporaire dans la perspective d’une transition vers une consultation « classique » tout en assurant la continuité des soins.

### Travail collaboratif intra et extra hospitalier

Afin de répondre à cette vocation d’accueil des personnes les plus précaires, les PASS doivent travailler en collaboration étroite avec les partenaires extérieurs ancrés sur le terrain, par exemple avec les équipes mobiles « santé précarité », les équipes spécialisées de « soins infirmiers précarité », les équipes mobiles de « psychiatrie-précarité », les maraudes, mais aussi avec les CPAM, les centres d’aide sociale à l’enfance, les centres de protection maternelle et infantile, les centres médico-sociaux locaux, le service intégré d’accueil et d’orientation, les Espaces solidarité insertion, les centres d’hébergement…

En parallèle, la PASS dédiée hospitalière a pour rôle de sensibiliser les professionnels hospitaliers à la question de la précarité, au repérage des personnes en situation de vulnérabilité et à l’orientation vers les structures adaptées, notamment vers les dispositifs des PASS. À ce titre, le cahier des charges de l’instruction de la DGOS de 2022 rappelle notamment l’importance de la coordination entre les structures des urgences et les PASS pour éviter que des patients ne renoncent aux soins en ne se rendant pas aux urgences, ou au contraire reviennent aux urgences de manière itérative pour des motifs ne relevant pas des urgences. Il est mentionné la nécessaire mise en place de protocole d’identification et d’orientation des patients précaires et la coordination médecin/infirmier diplômé d’État/PASS/travailleurs sociaux avec l’équipe soignante des urgences.

### Difficultés d’accès aux soins

En dépit des moyens mis en œuvre pour intégrer au système de soins les personnes les plus précaires, des freins à l’accès aux soins ont été identifiés.

Tout d’abord, les personnes en grande précarité, ne bénéficiant pas d’un hébergement stable ni d’un accès certain à la nourriture, relèguent au second plan les problématiques médicales. Pour elles, l’urgence concerne l’accès aux besoins physiologiques: s’alimenter, accéder à l’eau pour s’hydrater et assurer son hygiène corporelle, se mettre à l’abri pour assurer une protection thermique satisfaisante et sa sécurité. Il existe ainsi un parallélisme fort entre l’accès à un logement stable et la démarche d’aller vers le système de soins [17].

De cette situation précaire découle une temporalité differente qui cadence la vie des personnes sans-abri. La nécessité de se concentrer sur la survie quotidienne rend difficile l’établissement et le déploiement de projets à plus à moins long terme. Prévoir, organiser et honorer des rendezvous médicaux à un horaire et un jour précis peut sembler inadapté à ces personnes en grande précarité. Les PASS proposent le plus souvent un accueil sans rendez-vous pour tenter de pallier ce premier écueil.

Si elles sont très éloignées du système associatif sanitaire et social, les personnes en situation de précarité ne disposent pas nécessairement d’une connaissance claire et d’une compréhension satisfaisante des structures d’accueil dédiées aux personnes sans ressources financières. Par méconnaissance de l’entière prise en charge des frais liés aux soins, ces personnes s’interdisent l’accès aux consultations médicales, parfois jusqu’à ce que leur situation se dégrade au point de devoir s’orienter vers les services d’urgence. Concernant cet aspect financier, on peut citer l’absence de titres de transport ou la difficulté à se déplacer vers des structures médicales éloignées. Ces contraintes économiques, sociales et géographiques isolent davantage les populations précaires des systèmes de soins.

La méconnaissance de la langue française constitue un obstacle majeur pour les populations migrantes en situation de précarité. Faute de services d’interprétariat ou de médiation linguistique, ces personnes peinent à comprendre les informations médicales ou administratives essentielles, ce qui limite leur recours aux soins. Ces patients ignorent que les PASS ont accès à des services d’interprétariat.

Certaines personnes en situation de précarité et notamment en situation irrégulière sur le territoire, font preuve de méfiance envers les intervenants extérieurs. Non seulement ces personnes peuvent limiter leurs déplacements par crainte d’être interpellées ou contrôlées, notamment lorsqu’elles doivent se rendre dans des structures médicales, mais elles peuvent craindre également une instrumentalisation de leur situation médicale à des fins administratives, plus particulièrement dans les cas de délivrance par la préfecture d’une Obligation de quitter le territoire français (OQTF), ou dans le cadre d’une demande d’asile.

Le relais par les équipes mobiles de précarité et les accueils associatifs prend tout son sens dans l’orientation des personnes vulnérables vers les structures des PASS.

### Financement des PASS

Le financement des PASS provient du budget de l’État et la gestion est assurée par les CPAM, les ARS et les directions d’hôpitaux.

Avant 2022, les financements des structures de PASS relevaient de la Mission d’intérêt général (MIG). Ce dispositif permettait de financer directement les PASS *via* une dotation nationale inscrite dans le cadre des missions d’intérêt général et d’aide à la contractualisation des établissements de santé. Les crédits MIG étaient financés par une enveloppe dédiée, répartie par la DGOS, et allouée selon les directives nationales par les ARS, aux établissements de santé.

Ainsi, les PASS recevaient une dotation dite « MIG PASS » destinée aux frais des soins ambulatoires non recouvrables délivrés en leur sein, aux activités de pilotage, de coordination et d’évaluation. Les frais d’hospitalisation et les consultations facturables étaient exclus. L’objectif de cette dotation était de « soulager » l’hôpital des frais supplémentaires de personnel ainsi que de tous les frais liés à l’activité de la PASS comme notamment les dépenses pharmaceutiques, les frais d’examens médicaux, les prestations d’interprétariat ou les frais de transport pour les PASS mobiles.

Jusqu’à 2022, le montant des dotations accordées aux PASS était de 50 000 € minimum à plus de 450 000 € en fonction de la file active.

En 2022, la dotation totale allouée à la MIG pour les PASS s’est élevée à 88 904 676 € [11].

En 2022, dans le cadre du projet de loi de financement de la Sécurité Sociale, ces crédits ont été transférés au Fonds d’intervention régional (FIR). Ce changement visait à simplifier et territorialiser le financement, en permettant aux ARS de gérer ces fonds directement tout en réduisant le nombre de MIG (au nombre de 127 en 2021) et en rendant plus lisibles leurs objectifs. Les 88 904 676 € de fonds débloqués au titre de la MIG PASS en 2022 ont été réévalués et intégrés au FIR au titre d’un intérêt de leur territorialisation. Cette nouvelle organisation au sein du FIR s’inscrit dans une tentative de rationalisation budgétaire. Son efficacité réelle en termes de réduction des inégalités d’accès aux soins reste conditionnée à la capacité à venir des ARS à adapter concrètement leurs action aux besoins spécifiques des publics précaires, tout en préservant les moyens nécessaires au bon fonctionnement des différentes PASS.

La priorité mise en exergue par la mesure 27 des accords du « Ségur de la santé » de 2020 pour la lutte contre les inégalités sociales et territoriales de santé [13] a permis d’allouer des crédits pérennes et conséquents aux MIG PASS et MIG Précarité dans un effort de réduction des inégalités d’accès aux soins. Leur regroupement et leur intégration au sein du FIR a pour objectif de déployer une approche plus globale des enjeux de la précarité dans son ensemble à l’échelle régionale.

Les crédits du FIR, comme les MIG, sont soumis au principe d’annualité budgétaire. Cependant, les ARS peuvent, soit *via* le contrat pluriannuel d’objectifs et de moyens des établissements de santé, soit *via* un conventionnement spécifique, donner une visibilité pluriannuelle aux établissements et aux professionnels.

Le montant du financement FIR pour l’année 2023 s’est élevé à 105 milliards d’euros, et la dotation ARS en 2023 pour l’Île-de-France seule s’est élevée à 30 613 853 €.

### Activité chiffrée des PASS

D’après les données issues du pilotage des rapports d’activité des missions d’intérêt général, 206 562 personnes différentes ont consulté au moins une fois en 2021 dans une PASS et 252 778 en 2023, contre 193 108 en 2018. L’Île-de-France représente plus d’un cinquième de cette file active (53 330 personnes).

En 2023, les PASS dénombraient 64% de nouveaux patients dans leur file active (63% pour l’Île-de-France). La moyenne de consultations médicales en Île-de-France était de 2,08 consultations par patient, contre 1,95 en 2022.

Le rapport d’activité 2023 des PASS hospitalières d’Île-de-France établi par l’ARS faisait état d’une moyenne en file active de 615 patients par professionnel médical [1].

**Figure 2:**
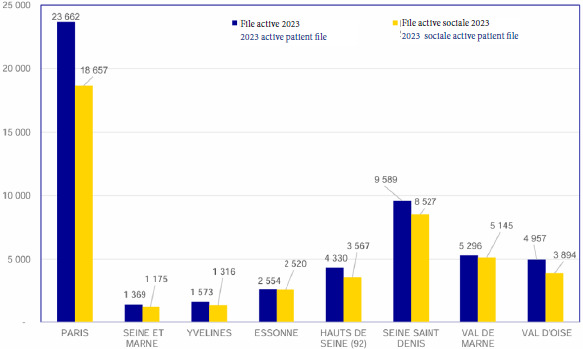
Activité sociale en Île-de-France en 2023, selon les données de la DGOS, rapportant le nombre de patients en file active des travailleurs sociaux dans les PASS d’Ile-de-France

Source: restitution de la DGOS de la Journée Régionale des PASS 2025

**Figure 3:**
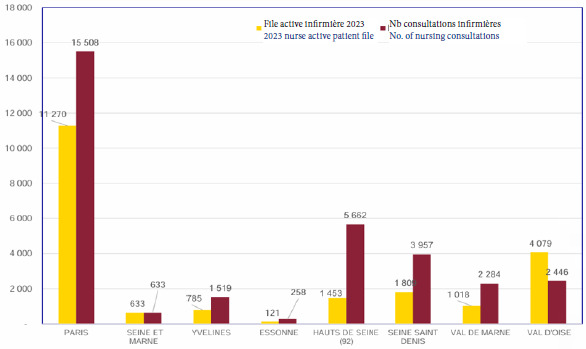
Activité infirmière en Île-de-France en 2023 selon les données de la DGOS, rapportant le nombre de patients en file active des infirmier(e)s dans les PASS d’Île-de-France et le nombre de consultations réalisées

Source: restitution de la DGOS de la Journée Régionale des PASS 2025

L’activité sociale en 2023 était de 1,71 par patient, pour une file active de 44 801 patients (Fig. 2). L’activité infirmière en Île-de-France faisait état de 1,52 consultations par patient, c’est-à-dire un total de 32 267 consultations en 2023 (Fig. 3).

### Profil des patients

La population est majoritairement masculine (57%) et jeune (1/3 des patients a moins de 25 ans). En 2023,8 029 mineurs non accompagnes (MNA) ont été pris en charge sur les PASS en France. On entend par « mineurs non accompagnés », les personnes se déclarant mineures, mais n’ayant pas encore été reconnues comme telles par l’État. En 2023, Le ministère de la Justice a effectivement reconnu le statut de mineur pour 19 370 d’entre eux.

En Île-de-France, 2 042 MNA déclarés ont été reçus, regroupant ainsi 25% des MNA accueillis dans les PASS du territoire. La part des MNA parmi les demandeurs d’asile reste stable, représentant environ 4% de l’ensemble des demandes déposées. Leur précarité de logement est manifeste: seuls 21% ont un logement fixe, 26% sont hébergés par des tiers et plus de 50% sont en hébergements très précaires. Leur absence de couverture maladie (56%) est un autre reflet de leur vulnérabilité.

**Figure 4:**
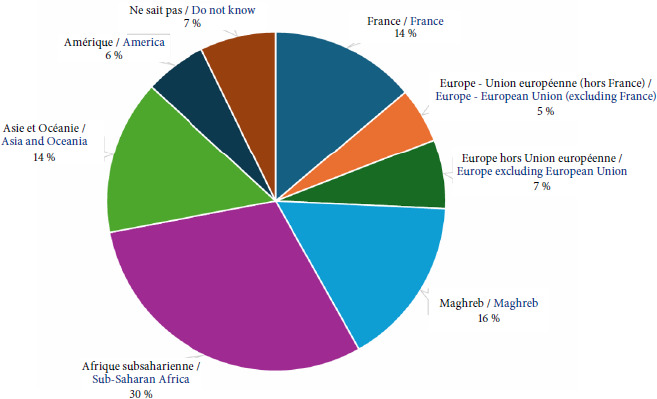
Origine géographiques des patients des PASS en France sur l’année 2023.

Source: restitution de la DGOS de la Journée Régionale des PASS 2025

L’augmentation de la part de la population issue de l’immigration est constante. En 2023, la France comptait 7,3 millions d’immigrés, représentant 10,7% de la population totale, marquant ainsi une augmentation de 3,8% par rapport à 2022 et de 32% par rapport à 2010 [6]. Ainsi, 74% des personnes accueillies dans les PASS sont des migrants dont 30% sont originaires d’Afrique subsaharienne (Fig. 4).

Les PASS ont su s’adapter et répondre aux demandes de ces patients issus de l’immigration qui représentaient originellement moins de 40% des accueillis.

Le recours à l’interprétariat professionnel au cours des consultations augmente au fil des ans. Plus de 65 000 actes d’interprétariat supplémentaires ont été réalisés en 2023, comparativement à l’année précédente, sur les PASS du territoire.

Le nombre de personnes allophones dans la file active totale des PASS en 2023 était de 58 507 en 2023 (contre 55 641 en 2022) soit environ un patient sur quatre. Les PASS de Martinique et de Guyane sont particulièrement concernées avec un taux respectif de 44% et 45% de patients allophones, tandis qu’en Île-de-France le taux de consultations en langue étrangère est de 21%, totalisant 10 205 actes d’interprétariat.

**Figure 5:**
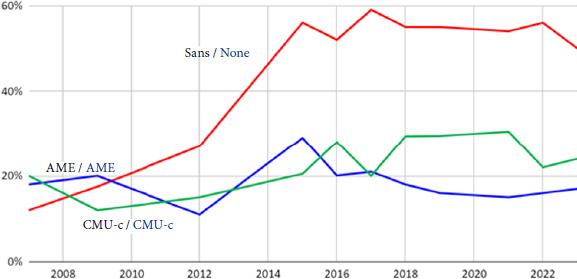
Évolution entre 2008 et 2022 de la part de patients de la PASS de l’Hôtel-Dieu bénéficiant de l’AME, de la CMU-c/CSS et ne bénéficiant d’aucune ouverture de droits.

Source: Statistiques internes de la PASS de l’Hôtel Dieu

En parallèle à la demande croissante de consultation, la réforme de la couverture santé mise en place au 1^er^ janvier 2020 a introduit plusieurs mesures qui ont complexifié l’accès aux soins pour les personnes étrangères précarisées, qu’elles soient en situation régulière ou irrégulière: réduction de la période de maintien des droits, délai de carence de trois mois concernant les demandeurs d’asile pour l’accès au régime général d’assurance maladie, délai de présence de trois mois requis pour toute demande d’AME, obligation de dépôt physique en CPAM pour toute première demande d’AME (sauf dérogations particulières de PASS hospitalières). Ces modifications ont eu pour effet de restreindre l’accès aux soins des populations étrangères précarisées, exacerbant leur vulnérabilité et augmentant la pression sur les dispositifs spécifiques comme les PASS (Fig. 5).

Dans la grande majorité des cas, les soins délivrés par la PASS ne sont pas soumis à facturation pour les patients. L’évaluation sociale par les assistants sociaux peut faire apparaître que, au moment de la réalisation des actes, les patients ne pourront pas bénéficier d’une couverture sociale. Leur prise en charge bascule alors dans le budget PASS.

En 2023,69% des patients reçus dans les PASS réparties sur tout le territoire ne disposaient d’aucune couverture maladie, représentant 152 805 patients au total; 10% des 221 457 patients des files actives disposaient de l’AME et 10% d’une protection universelle maladie (PUMA) sans complémentaire.

La précarité des patients reçus dans les PASS se caractérise également par leurs faibles ressources financières: 72% des patients des PASS hospitalières en France ne bénéficient d’aucune ressource, ce qui rend l’accès à l’hébergement difficile, notamment si les patients ne relèvent pas des droits aux conditions matérielles d’accueil dans le cadre d’un droit d’asile. Ainsi, le rapport DGOS 2023 fait état de 27% de patients vivant à la rue ou bénéficiant ponctuellement de l’accès à un hébergement d’urgence *(via* le numéro d’urgence sociale 115 majoritairement) et 2% vivant dans des squats ou des bidonvilles; 36% des patients sont logés par des amis ou des proches et 13% par des associations sur une période prolongée. Seulement 13% déclarent bénéficier d’un hébergement fixe et stable.

## Les PASS de l’Hôtel-Dieu de Paris: « *Medicus et hospes,* hôte et médecin »

L’Hôtel-Dieu a été fondé vers 660 par Saint Landry, officier à la chancellerie royale sous Clovis II. Il y recevait, à ses propres dépens, non seulement les malades mais aussi les mendiants et les simples pèlerins. « *Medicus et hospes,* hôte et médecin », telle était la devise de Saint Landry, nommé Évêque de Paris en l’an 630.

Symbole dès sa création d’hospitalité et de charité, l’hôpital a conservé ces valeurs qui demeurent encore aujourd’hui très présentes dans son fonctionnement. Sa situation géographique au cœur de la capitale, au croisement des RER, fait de l’Hôtel-Dieu un hôpital de proximité pour de nombreux habitants des banlieues, au même titre que pour ceux qui travaillent ou résident à Paris.

### Personnels et services concernés

La PASS de ¡’Hôtel-Dieu a fait le choix de faciliter au maximum l’accueil des patients en situation de précarité en organisant dans une unité de lieu une prise en charge multidisciplinaire. Forte de ses 10 médecins (correspondant à deux équivalents temps-plein), dont une oncologue, elle compte 3 infirmiers, 3 assistants sociaux, une secrétaire sociale et une aide-soignante. Elle participe à la formation et à la sensibilisation des professionnels du secteur sanitaire et social en accueillant en son sein six internes de médecine générale par semestre et quatre externes par trimestre, huit jeunes volontaires en roulement sur l’année, quatre stagiaires infirmiers et deux stagiaires des services sociaux. Les jeunes volontaires ont pour mission d’accompagner les patients dans leurs démarches avant et après les rendez-vous médicaux, mais aussi au sein des differents services. À ces effectifs de la PASS de médecine générale, s’ajoutent ceux des PASS ophtalmologique et bucco-dentaire avec l’intervention d’une médecin ophtalmologue bénévole, trois dentistes sur site et trois orthoptistes.

Le service de radiologie du site de l’Hôtel-Dieu vient compléter le panier de soins en offrant un plateau technique privilégié pour les patients de la PASS qui peuvent en bénéficier au même titre que les assurés sociaux.

La PASS bénéficie également d’une offre podologique via le dispositif PODOPASS, grâce à une podologue bénévole qui intervient de manière hebdomadaire pour des soins qui sont difficiles d’accès pour des considérations financières et qui sont pourtant nécessaires, particulièrement chez les patients précaires à risque de complications ostéo-articulaires et infectieuses.

En 2016, devant la très grande difficulté à accéder aux soins ophtalmologiques de ces patients, compte tenu de la faible démographie médicale, des soins coûteux délivrés en secteur 2 et du prix élevé des lunettes, une PASS ophtalmologique a été créée, avec la possibilité de fournir les lunettes *in situ* grâce à un partenariat avec One Sight Essilor Luxottica.

En 2018, une PASS dentaire est venue compléter l’offre de soins proposée par la PASS généraliste, en raison des besoins importants exprimés par les patients dont la prise en charge en ville était freinée par des raisons identiques à celle de la prise en charge ophtalmologique. Seuls les soins prothétiques n’y sont pas pratiqués. Une réflexion est en cours sur ce sujet.

Plus récemment, nous avons constaté que les soins de kinésithérapie sont devenus inaccessibles: même avec une couverture sociale solidaire (CSS, ancienne couverture médicale universelle complémentaire CMU-c) ou avec une AME, nombre de patients n’arrivent pas à être pris en charge. Beaucoup disent être refusés par le praticien. D’autres révèlent qu’un supplément financier leur a été demandé, supplément qu’ils ne peuvent pas prendre à leur charge. Aussi, depuis 2021, la PASS de l’Hôtel-Dieu, grâce à un partenariat avec Rééducateurs solidaires, peut offrir *in situ,* via son dispositif « KinéPass », ces soins qui sont indispensables, comme en témoigne la fréquence des motifs traumatologiques.

Depuis 2022, la collaboration étroite avec le service de psychiatrie et le soutien de la Fondation de France ont permis le renforcement de la prise en charge psychiatrique des patients en situation de vulnérabilité, avec la mise en place du dispositif COMPASS-PSY. Il s’agit d’un dispositif en 4 axes liés à la très forte prévalence des troubles psychiques dans cette population. Premièrement, des *staffs* psychiatriques hebdomadaires avec une psychologue et un psychiatre permettent aux soignants de poursuivre les soins dans l’axe des conseils et décisions prises, d’orienter les patients de manière adaptée et de se former. Deuxièmement, des consultations en binôme médecin/psychologue dites « de compagnonnage » permettent aux soignants d’acquérir un savoir-faire et un savoir-être face aux demandes psychiatriques, et en particulier aux troubles de stress post-traumatique, et apportent aux patients un apaisement et une écoute bienveillante. Des ateliers de groupe hebdomadaires de patients axés sur la stabilisation émotionnelle complètent l’offre. Enfin, des consultations dédiées avec un psychiatrique peuvent être proposées.

La possibilité d’offrir un lieu de soins et une prise en charge holistique dans plusieurs permanences d’accès aux soins dans un même établissement (plusieurs PASS: PASS généraliste, PASS buccodentaire, PASS ophtalmologie, KinéPASS, COMPASS-PSY) facilite la compréhension du parcours de soin par les patients et leur adhésion aux soins. À titre d’exemple, le dépistage systématique chez les nouveaux patients, notamment des maladies infectieuses, peut se faire au sein de la même structure grâce aux actes infirmiers et aux radiographies thoraciques qui peuvent se faire le jour même après la consultation médicale. Les traitements peuvent être ensuite délivrés par la pharmacie hospitalière.

La PASS bénéficie du soutien d’associations, notamment des Amis de la PASS, qui participent à faciliter, par des moyens humains et financiers, l’accès aux soins des patients les plus vulnérables accueillis au sein de l’Hôtel-Dieu.

### Activité globale

L’accueil des plus démunis se décline sur l’ensemble de l’hôpital mais la présence d’une PASS généraliste depuis 1998 a grandement facilité leur prise en charge: en 2023, plus de 10 500 consultations médicales, 3 200 entretiens sociaux et 4 500 actes infirmiers ont été délivrés pour 4 144 patients différents (file active). En 10 ans, la PASS a effectué plus de 100 000 prises en charge médico-sociales. La PASS de l’Hôtel-Dieu a comptabilisé 75% de nouveaux patients dans sa file active en 2023. En 2023,844 consultations ophtalmologiques été dispensées chez 691 patients. La PASS dentaire a fourni 1 330 consultations pour 707 patients en 2023.

### Profil socio-démographique des patients de la PASS de l’Hôtel-Dieu

**Figure 6:**
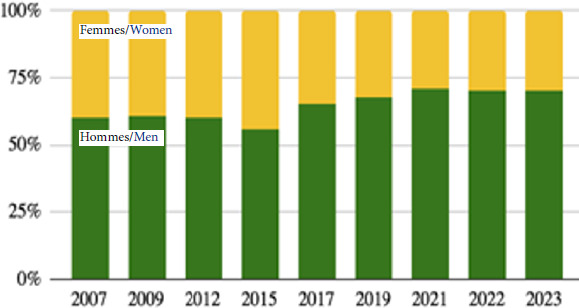
Évolution du rapport homme/femme chez les consultants de la PASS de l’Hôtel-Dieu de 2007 à 2023

Source: Statistiques internes de la PASS de l’Hôtel Dieu

De 1998 à 2015, la proportion des femmes consultant à la PASS de l’Hôtel-Dieu a augmenté, reflétant la part croissante des femmes dans la migration subsaharienne (> 40%). Depuis 2015, la tendance s’est inversée et la proportion des hommes n’a cessé de croître, passant de 56% à 71%. Ce pourcentage, différent de la moyenne nationale, reflète la forte fréquentation de migrants originaires d’Asie, essentiellement d’hommes afghans (Fig. 6).

**Figure 7:**
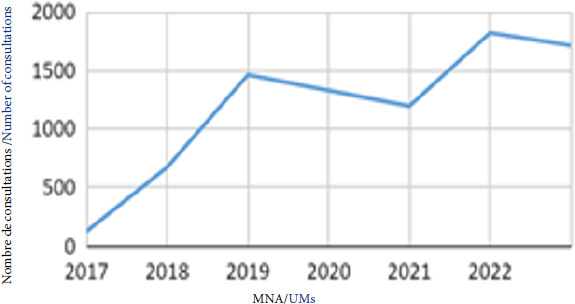
Évolution du nombre de consultations dédiées à des mineurs non accompagnés (MNA) entre 2017 et 2023 au sein de la PASS de l’Hôtel-Dieu.

Source: Statistiques internes de la PASS de l’Hôtel Dieu

La moyenne d’âge a chuté de près de 10 points sur les 8 dernières années, passant de 43 à 34 ans. Ce constat s’explique par l’augmentation croissante de consultations dédiées aux très jeunes migrants, mineurs non accompagnés (MNA). En 2023, la PASS de l’Hôtel-Dieu a effectué 1 714 consultations (> 15% de la file active) pour des MNA contre 50 en 2015 (Fig. 7). Cette situation est généralisable au reste de la France, puisque d’après le rapport sur les MNA du ministère de la Justice de mi-2023,15 000 MNA ont été identifiés en 2022 *versus* 5 000 en 2014 [9].

**Figure 8:**
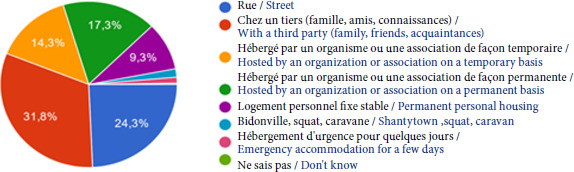
Répartition des différents types de domicile déclarés par les patients de la PASS de l’Hôtel-Dieu en 2023

Source: Enquête ponctuelle de 2023 sur 432 hétéro-évaluations

Les patients présentent tous un ou plusieurs facteurs de vulnérabilité, selon l’étude menée en 2023 et portant sur l’évaluation de la vulnérabilité et des conditions médico-sociales des patients [14]: • Un quart des patients reçus en consultation dorment dans la rue, 32% sont hébergés par un tiers, 32% sont accueillis par une association et moins de 10% disposent d’un logement personnel (Fig. 8).

**Figure 9:**
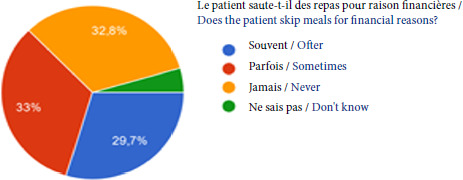
Difficulté d’accès aux repas par manque de ressources chez les patients de la PASS de l’Hôtel-Dieu en 2023

Source: Enquête ponctuelle de 2023 sur 432 hétéro-évaluations
- La moitié n’a pas de couverture maladie, 17% disposent de l’AME, 24% de la CSS et 10% bénéficient seulement d’un accès à la Sécurité sociale sans complémentaire (Fig. 5).
- Près de la moitié des patients a un suivi social en dehors de l’unité et 60% sont adressés par une association partenaire.

- Près des 2/3 n’accèdent pas à 3 repas par jour pour des raisons financières (Fig. 9).

**Figure 10:**
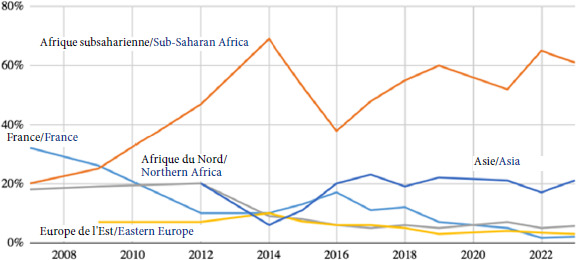
Répartition selon l’origine géographique des patients de la PASS de l’Hôtel-Dieu entre 2008 et 2022

Source: Statistiques internes de la PASS de l’Hôtel Dieu

Plus de 100 pays d’origine étaient représentés en 2023, avec une tendance à la hausse de cette diversité: 2% étaient originaires de France en 2023 *versus* 13% en 2015. Si la proportion des patients originaires d’Afrique subsaharienne est majoritaire (61% en 2023 *versus* 53% en 2015), la part des patients d’origine asiatique est croissante (21% *versus* 11%), alors que celles du Maghreb (6% *versus* 8%) ou de l’Europe (3% *versus* 7%) demeurent stables (Fig. 10).

**Figure 11:**
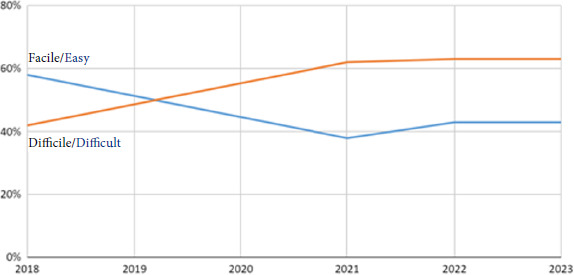
Évaluation de la difficulté de communication par le médecin au cours de la consultation avec le patient au sein de la PASS de l’Hôtel-Dieu et son évolution entre 2018 et 2023.

Source: Statistiques internes de la PASS de l’Hôtel Dieu

**Figure 12:**
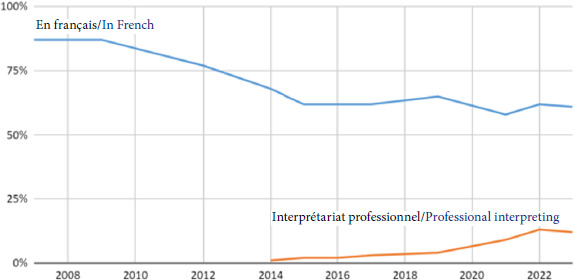
Évolution du recours à l’interprétariat professionnel entre 2008 et 2023

Source: Statistiques internes de la PASS de l’Hôtel Dieu

Concernant la langue utilisée par les patients, les statistiques concordent avec ce qui est observé en Île-de-France [16]: 21% des consultations qui ont eu lieu en 2023 ont été menées en langue étrangère. La langue la plus représentée était le Pachto (Afghanistan et Pakistan), suivi du Dari (Afghanistan), puis du Bengali (Bangladesh, Inde). Pour 60% des consultations, la communication a été jugée difficile par le médecin (Fig. 11), ce qui explique, en partie, l’explosion des demandes de recours à l’interprétariat professionnel: 1 244 appels soit 21% des consultations *versus* 4% en 2018 (Fig. 12).

### Pathologies observées

**Figure 13:**
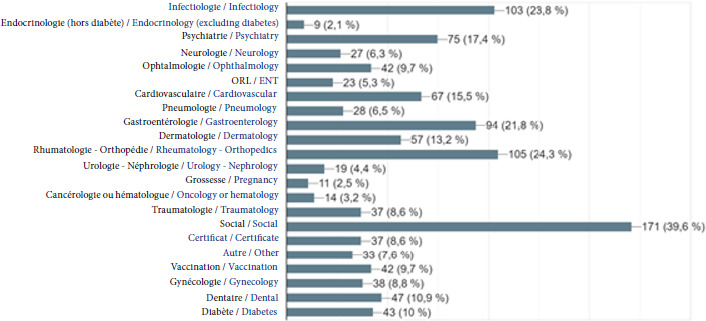
Diagnostic(s) retenu(s) en fin de consultation par le médecin de la PASS de l’Hôtel-Dieu

Source: Enquête ponctuelle de 2023 sur 432 hétéro-évaluations

Les motifs de consultation sont très divers. Des tendances générales se dessinent néanmoins: les motifs ayant trait à la traumatologie sont surreprésentés, de même que les pathologies infectieuses. Les motifs relatifs à la psychiatrie et à la gastro-entérologie (1 patient sur 4) sont plus fréquents qu’en population générale. En revanche, les consultations pour viroses sont très peu représentées (5%) (Fig. 13).

**Figure 14:**
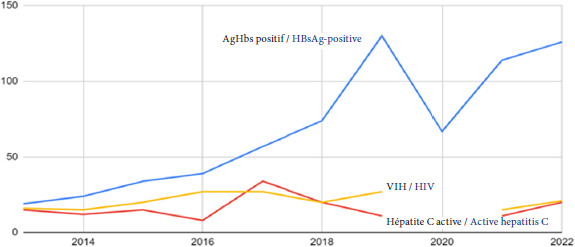
Estimation du nombre d’AgHbs positif, du nombre d’hépatite C active (donnée manquante 2020) et du nombre de VIH (donnée manquante 2020) par an au sein de la PASS de l’Hôtel-Dieu

Source: COPIL de la PASS de l’Hôtel-Dieu 2023
- **Hépatite virale C.** L’hépatite C active (définie par une sérologie et une charge virale positive) a une prévalence de 1,14% parmi les patients de la PASS en 2022, soit 4 fois plus que dans la population générale adulte en France métropolitaine: en 2016, le baromètre santé estimait sa prévalence à 0,3% (IC95%: 0,13 - 0,70) [15] (Fig. 14).
- **Infection par le VIH.** Le taux d’infection à VIH est établi à 1,17% en 2022. Cette donnée est à surveiller au cours des prochaines années. En effet, d’après Santé publique France, le nombre de découvertes de séropositivité VIH a augmenté entre 2021 et 2023, particulièrement chez les personnes nées à l’étranger [16] (Fig. 15).

Les pathologies les plus fréquentes ayant motivé une consultation en 2023 se déclinent comme suit:

- **Hypertension artérielle (HTA).** Le taux d’HTA est de 19,2%. L’HTA touche particulièrement les plus de 40 ans (44%). La découverte d’HTA se fait souvent à des grades sévères (> 180/110mmHg de pression). Pour rappel, en France métropolitaine, près d’un adulte sur 3 est hypertendu, surtout dans les classes d’âge élevées. La différence entre la population générale et la population de la PASS concerne l’atteinte plus précoce et plus sévère de l’HTA chez cette dernière.
- **Diabète.** Le diabète concentre 12,6% des motifs de consultation avec 10% de diabètes non insulino-dépendants et 2,6% de diabètes insulino-dépendants. Ce sont 29,9% des plus de 40 ans qui sont touchés, soit presque 1 patient sur 3 [14]. Pour rappel, en France, la prévalence de diabète est de 5,6% [18]. Les patients ont un taux d’HbA1c plus élevé que la moyenne nationale, un suivi moins régulier et des microangiopathies plus fréquentes [7].
- **Hépatite virale B.** L’hépatite B est particulièrement prégnante avec un taux, en 2022, de 9,9% (IC95%: 7,5 - 12,3). Pour rappel, la prévalence en France métropolitaine en 2016 était estimée à 0,3% (0,13 - 0,70) [15] (Fig. 14).

**Figure 15:**
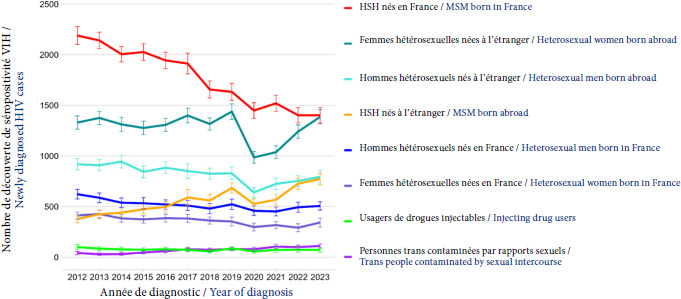
Évolution du nombre de découverte de séropositivité VIH, en France, selon le type de population (définie par le genre, le mode de contamination probable et le lieu de naissance) entre 2012 et 2023. HSH: Hommes ayant des relations sexuelles avec des hommes

Source: Déclaration obligatoire de l’infection à VIH (DO VIH), Données au 30/06/2024, corrigées pour les délais de déclaration, la sous-déclaration et les valeurs manquante
- Tuberculose. Chaque année, une trentaine de diagnostics de tuberculose maladie sont posés sur les 4 000 patients différents reçus. De manière plus factuelle, on peut citer les données de la Société de pathologie infectieuse de langue française: l’incidence chez les personnes nées hors de France (31/100 000) est environ 10 fois supérieure à celle des personnes nées en France. Elle affecte plus particulièrement les strates les plus pauvres de la population, notamment les personnes sans domicile fixe chez qui l’incidence (autour de 61/100 000) dépasse de très loin celle des autres groupes sociaux [19].
- Infections tuberculeuses latentes. Trois cents tests de libération de l’interféron gamma ont été réalisés en 2022 au sein de la PASS auprès des patients mineurs et migrants venant de zones de forte prévalence, afin d’identifier les cas de tuberculose latente: 35% des test réalisés se sont avérés positifs. Comparativement, 23% de la population mondiale en 2014 avaient une tuberculose latente. La question du dépistage et du traitement des infections tuberculeuses latentes présente un enjeu majeur de santé publique, compte tenu du risque de multiplication de cas émergents de tuberculose maladie, véhiculés par des personnes en situation de migration, surtout dans les pays à faible incidence de tuberculose [4]

### Vaccinations

En 2022,2 700 vaccinations ont été réalisées à la PASS de l’Hôtel-Dieu dans le but de rattraper un retard vaccinal selon le calendrier des recommandations françaises. Les principaux vaccins qui ont été administrés étaient:

- le vaccin conjugué contre la diphtérie, le tétanos, la poliomyélite et la coqueluche;
- le vaccin contre la rougeole, les oreillons et la rubéole;
- le vaccin contre l’hépatite B.

Les autres vaccinations réalisées à la PASS ont concerné la méningite ACWY (pour les moins de 25 ans), le pneumocoque (selon les indications), l’hépatite A (pour les patients ayant une infection par le VHB essentiellement) et la Covid19. La vaccination contre le HPV (Papillomavirus humain) n’est pas encore proposée, pour des raisons de coût.

### Prise en charge psychologique et psychiatrique

Cette activité est croissante, la plupart des patients vivant avec une souffrance morale ou présentant des syndromes de stress post-traumatique en lien avec leur histoire passée ou en rapport avec leur parcours migratoire. Leur prise en charge est pluridisciplinaire, complexe et nécessite un suivi rapproché. Une personne sur cinq (22%) ayant connu la guerre ou une autre situation de conflit il y a 10 ans ou moins souffre de dépression, d’anxiété, de stress post-traumatique, de troubles bipolaires ou de schizophrénie [2]. Le personnel soignant est sensibilisé et formé à la prise en charge de ces affections. Cette prise en charge adaptée fait de la PASS un lieu privilégié d’accueil pour les personnes en situation de grande vulnérabilité psychique et psychiatrique.

## Discussion

Le mode de fonctionnement d’une PASS étant extrêmement différent d’un établissement à l’autre, il est difficile de comparer leur activité. L’exemple de la PASS de l’Hôtel-Dieu permet cependant d’identifier des contraintes propres à l’ensemble des PASS.

Depuis sa création, l’activité de la PASS de l’Hôtel-Dieu n’a fait que croître, tout comme celles des autres PASS. Le profil des patients et leur origine géographique ont évolué au gré des années et des conflits géopolitiques. Aujourd’hui, la part de personnes issues de l’immigration prend une place majeure et leur état de santé justifie souvent un suivi médical urgent et régulier.

La complexité de la prise en charge de personnes malades et en situation de fragilité sociale rend les actions menées longues, chronophages et efficaces seulement sur le temps long.

La complexité de cette prise en charge pourrait encourager les disparités sociales quant à l’accès aux soins de ces patients et favoriser la résignation ou l’indifférence des professionnels de santé qui ne disposeraient pas de la formation, des moyens et de la structure nécessaires pour accompagner ces patients. Les PASS ont pour mission d’aller vers ces patients en grande vulnérabilité, d’identifier les écueils dans leur accès aux soins et de les réintégrer vers le système sanitaire avant de les accompagner vers les structures non spécifiques. Les personnes les plus vulnérables révèlent des dynamiques sociales et des dysfonctionnements structurels qu’il est impératif d’analyser et de comprendre. La prise en charge de l’être humain dans le cadre des soins ne peut se limiter à la seule dimension technique, bien que celle-ci soit indispensable et indéniablement bénéfique. L’approche médicale globale met en évidence l’importance capitale de la relation interhumaine qui joue un rôle complémentaire et essentiel dans le processus de guérison. Cette perspective holistique considère l’individu dans sa globalité, intégrant les dimensions physiques, émotionnelles, mentales, sociales et spirituelles, de manière à favoriser un équilibre harmonieux et durable entre ces différentes composantes.

Les PASS, dont celle de l’Hôtel-Dieu, sont particulièrement confrontées aux changements de la société. Selon Xavier Emmanuelli, « le monde s’est mis en marche et nous ne pourrons pas l’arrêter ». Nous n’entrerons pas ici dans les débats politiques sur la migration. C’est plutôt le serment d’Hippocrate, ligne rouge qu’il convient de réentendre particulièrement aujourd’hui: « Je respecterai toutes les personnes, leur autonomie et leur volonté, sans discrimination. J’interviendrai pour les protéger si elles sont vulnérables ou menacées dans leur intégrité ou dans leur dignité… ». Face à son patient, le médecin n’est pas là pour juger du bien-fondé ou non des politiques migratoires. Il est face à une personne souffrante qu’il convient de soulager par tous les moyens possibles.

Les PASS, après 25 ans d’existence, ont démontré leur valeur ajoutée dans le système de soins. Elles accueillent des patients en situations sociale et médicale complexes et leur proposent une prise en charge médico-sociale adaptée, individualisée, globale. Elles constituent une alternative aux consultations aux urgences et au « *zapping* » médical de cette population. Elles sont un reflet de l’évolution de la société française et doivent en permanence s’adapter à la demande qui change au gré de l’histoire mondiale. Preuve en est leur rôle auprès des mineurs étrangers isolés non accompagnés livrés à eux-mêmes sur notre territoire. Elles sont un observatoire mal exploité de l’état de santé d’une frange de la population. Elles sont et doivent rester avant tout un lieu de soins.

Ces dispositifs ne peuvent exister sans un soutien financier étatique fort, sans un soutien local (ARS, direction et encadrement hospitalier) et sans un engagement professionnel adapté car ils sont par essence fragiles, non-rentables à court terme, interrogeant par la population qu’ils drainent, dérangeant par l’organisation spécifique qu’ils demandent.

### Spécificité de la prise en charge des plus vulnérables

Forte de son expérience de 25 ans auprès des plus démunis, la PASS de l’Hôtel-Dieu a identifié plusieurs constats à prendre en compte dans l’établissement des grands principes de la prise en charge de personnes vulnérables:

- Plus les patients sont démunis, plus la prise en compte de la dimension sociale devient un impératif dans la mise en place du parcours de soins et le choix thérapeutique. La précarité sociale prend toute la place au détriment de la dimension médicale, il faut savoir la prendre en compte en premier lieu.
- Plus les patients sont vulnérables socialement et culturellement différents, plus il est nécessaire d’adapter notre dispositif et notre accueil à leur service pour faciliter leur accès aux soins.
- La perte de repères spatio-temporels des personnes sans domicile fixe est une des clés de compréhension des « rendez-vous manqués » dont nous devons tirer les conséquences: l’unité de lieu et de temps des soins sont des prérequis indispensables. L’accueil sans rendez-vous vient renforcer l’adaptation de la structure à la temporalité des personnes en situation de grande précarité.

Le rôle des PASS est de développer les moyens permettant des soins optimaux pour ces personnes en situation de précarité, en tendant vers un résultat optimal mais en se détachant de celuici tant le « succès » est rare.

### Grands principes de la prise en charge médicale des personnes en situation de vulnérabilité

#### Repérer les facteurs de vulnérabilité

Les principaux facteurs de vulnérabilité à repérer sont les suivants:

- **Le logement:** en France, 15 millions de personnes étaient considérées en fragilité de logement en 2023 dont 4,1 millions « non ou mal logées » [3]. Lors de l’interrogatoire, il est utile de rechercher dans quel quartier vit le patient, s’il dort dans la rue, combien de personnes logent dans la même chambre, s’il existe une contrepartie à l’hébergement, financière ou de l’ordre de tâches ménagères ou de faveurs sexuelles, s’il sollicite le « 115 », etc.
- **La précarité de logement** engendre des conséquences sur la santé: intoxications au monoxyde de carbone, au plomb, bronchites chroniques, asthme, tuberculose, arthrose, anxiété et dépression, céphalées, eczéma, infection dermatologique bactérienne, parasitaire et fongique, troubles du sommeil… Mieux identifier les conditions de vie permet d’optimiser le dépistage et la démarche thérapeutique adaptée au patient.
- **La précarité de l’emploi:** il en découle une insécurité financière, des difficultés d’hébergement, une rupture des liens sociaux (y compris avec la famille restée au pays), une vie sans rythme, une baisse d’estime de soi et une moindre reconnaissance sociale.
- **La précarité alimentaire:** en quantité elle peut provoquer une perte de poids alors qu’en qualité elle engendre plus souvent un surpoids et des carences (fer, folates, B12, vitamine C, protéines…).
- **La pauvreté financière:** elle s’évalue par la précarité de logement, d’emploi et la difficulté d’accès à l’alimentation.
- **L’isolement:** il est souvent aggravé par la barrière de langue et par la consommation de toxiques.
- **La précarité liée à la migration:** si « être migrant » n’est pas équivalent à « être en situation précaire », une attention particulière doit être portée aux personnes migrantes car nombre d’entre elles vivent aujourd’hui dans des conditions particulièrement difficiles. Ces patients peuvent être administrativement en situation irrégulière sans avoir la possibilité de travailler légalement et ils disposent de ressources financières limitées. La barrière linguistique et culturelle renforce leur isolement et leur mauvaise compréhension du système de santé et des réglementations régissant l’immigration, en perpétuelle évolution. La crainte liée à leur situation d’irrégularité, qui peut donner lieu à un renvoi dans leur pays, suscite la méfiance à l’égard des structures administratives mais aussi médicales, les incitant à s’éloigner davantage des structures de soins. Enfin, l’absence d’hébergement aggrave leur souffrance psychique liée à l’exil, au parcours migratoire, à la non-intégration ou aux violences subies.

#### Être à l’écoute de la souffrance en lien avec la précarité

L’écoute constitue une étape essentielle dans la démarche thérapeutique, tout particulièrement pour les populations précaires ou vulnérables. Elle permet de libérer une parole souvent négligée et de construire une alliance entre le soignant et le patient, primordiale pour garantir une prise en charge adaptée. Ce processus peut être comparé aux soins de confort (anciennement soins palliatifs) où l’objectif est d’améliorer la qualité de vie en tenant compte des besoins immédiats du patient. Dans les deux cas, l’écoute joue un rôle central pour répondre à des situations marquées par l’urgence et l’exclusion.

Dans un contexte de temporalité et d’immédiateté où les attentes des patients vis-à-vis du système de santé sont souvent pressantes et le suivi inconstant, l’écoute permet de dépasser les simples échanges fonctionnels pour établir une compréhension mutuelle. Cela aide non seulement le patient à se projeter dans le temps mais aussi à renforcer son adhésion aux soins. Pour les personnes en situation de précarité, cette démarche contribue à lutter contre l’exclusion sociale et médicale en intégrant leurs problématiques spécifiques dans la prise en charge globale, en proposant des solutions adaptées aux conditions de vie, des traitements optimaux et adaptés au contexte psycho-social. En outre, cette approche d’écoute active s’inscrit dans des démarches dites d’« aller-vers », particulièrement pertinentes pour les publics éloignés du système de santé afin de créer un lien et d’initier un suivi médical ou social. En cela, l’écoute devient un outil puissant non seulement pour établir une relation thérapeutique mais aussi pour réintégrer les populations précaires dans le parcours de soins, comme première étape de réinsertion.

#### Proposer une prise en charge médicosociale pluridisciplinaire et travailler en réseau

L’intrication médico-sociale des situations des patients en précarité impose une approche collaborative et multidisciplinaire. Travailler en partenariat avec les équipes sociales et, plus largement, avec les partenaires associatifs n’est pas simplement une valeur ajoutée, c’est une condition essentielle pour garantir l’efficacité de la prise en charge de ces patients. Ils présentent souvent des problématiques complexes mêlant santé physique, troubles psychiques, précarité sociale et difficultés administratives qui ne peuvent être résolues que par une collaboration étroite entre travailleurs sociaux, équipe soignante et partenaires associatifs.

Les assistants sociaux jouent un rôle clef dans la recherche de solutions concrètes pour les patients, telles que l’ouverture des droits médicaux, l’accès au logement, l’aide alimentaire ou vestimentaire ou encore le relais juridique. Leur intervention permet de répondre aux besoins fondamentaux des patients qui sont souvent des prérequis indispensables à leur adhésion aux soins médicaux. Les associations, quant à elles, apportent une expertise complémentaire et un soutien logistique pour des actions ciblées (hébergement d’urgence, accompagnement psychologique, médiation culturelle). Elles contribuent largement à réduire les barrières d’accès aux soins en offrant des services adaptés aux publics précarisés, et ce sont souvent elles qui identifient les besoins médicaux peu exprimés des patients et qui organisent le premier rendez-vous vers la PASS.

Cette collaboration transdisciplinaire vise à décloisonner les interventions médicales et sociales pour répondre efficacement aux besoins complexes des patients précaires et s’ancrer dans la démarche de la mesure 27 des accords du Ségur de la santé pour réduire les inégalités d’accès aux soins. Le dispositif PASS manifeste pleinement son rôle de passerelle par la réintégration dans le droit commun et l’autonomisation à long terme de ces patients vulnérables.

#### Seconder puis autonomiser

Le système de soin et les démarches administratives de l’immigration, malgré leur tendance à la simplification et à la dématérialisation, demeurent complexes à comprendre et à intégrer. Les patients sont, au début de leur prise en charge, très souvent dépendants. Une prise en charge optimale passe par la prise de leurs rendez-vous médicaux, de l’accompagnement physique vers les services de radiologie, la pharmacie…

L’exemple de l’organisation d’une colposcopie illustre ces difficultés: la patiente doit se rendre à plusieurs rendez-vous avec le spécialiste afin qu’il pose l’indication et explique la procédure, si possible dans sa langue native ou par traduction. La consultation doit détailler le déroulé de l’opération mais aussi celui de la préparation. Sur le plan organisationnel, il convient d’imprimer un plan qui permettra au patient de se rendre à l’hôpital où aura lieu l’intervention, différent parfois du lieu de consultation, et de s’assurer de la bonne compréhension du patient en le faisant répéter. À ces difficultés s’ajoutent la barrière de langue ou l’analphabétisme francophone.

L’étape de l’autonomisation vient dans un second temps: elle est progressive et jonchée de beaucoup de « ratés ». Le soignant doit alors persévérer sans culpabiliser et trouver des moyens de faire « avec » le patient.

#### Favoriser l’unité de lieu et de temps des soins

Les personnes en situation de grande précarité et de vulnérabilité ont souvent une capacité limitée à se projeter dans l’avenir, leur quotidien étant régi par la recherche urgente de logement et de nourriture.

Ce contexte impose un rapport à l’immédiateté où les besoins doivent être satisfaits « ici et maintenant ». Cette priorité donnée à l’urgence rend difficile toute démarche qui nécessite une vision à long terme.

C’est pourquoi, dans la mesure du possible, un suivi inscrit dans la durée est proposé pour accompagner ces patients vers une projection dans le temps. Offrir une continuité dans les soins permet de créer un cadre stable, essentiel pour favoriser leur reconstruction personnelle. L’unité de lieu et de temps dans la prise en charge, c’est-à-dire des points de repère fixes et réguliers, agit comme un ancrage qui donne au patient une base solide sur laquelle il peut commencer à se reconstruire progressivement, en dépassant la logique de survie immédiate.

#### Adapter le traitement et les conseils aux conditions de vie

Le nombre, les horaires, les modalités de prises du traitement doivent être raisonnés en fonction des conditions de vie, spécialement pour ceux à la rue. La difficulté dans laquelle les patients peuvent se trouver pour se rendre sur place doit être évaluée. La prise des médicaments et le choix des thérapeutiques ne peuvent se faire sans la connaissance préalable des conditions de vie et des contraintes du patient.

## Conclusion

La prise en charge des personnes nécessitant des soins médicaux et confrontées à des difficultés sociales constitue un défi complexe, intemporel, où les vulnérabilités médicales et sociales s’intriquent, limitant l’efficacité des interventions isolées. De nombreux problèmes persistent comme le financement des examens complémentaires, des médicaments et des hospitalisations ou comme les modalités d’intégration des patients au système de soin commun. Les PASS doivent relever le défi de soigner les personnes les plus fragiles, les plus éloignées du système de soins, en valorisant leur potentiel. L’absence de soins engendrerait des conséquences graves pour ces personnes comme pour la collectivité, tant sur le plan sanitaire (risques infectieux) que financier (coût des complications non traitées). L’accompagnement des populations vulnérables, en grande précarité, migrantes ou marginalisées ne relève pas d’un simple choix pour la société française mais d’un impératif éthique et humanitaire.

## Remerciements

Un immense merci au Pr Éric Caumes pour son soutien et ses conseils pour nos patients, un très grand merci au Pr Eric Pichard pour ses remarques si pertinentes.

## Sources de financement

Aucun financement industriel ou commercial n’a été reçu pour la réalisation de ce travail.

## Conflits d’intérêts

Aucun conflit d’intérêts n’a été déclaré.
