# Supplementary material for: PASS units: mechanisms for delivering healthcare to underserved populations. The example of the Hôtel-Dieu PASS, Paris
Source: Med Trop Sante Int. 2025 Aug 6;5(4):mtsi.v5i4.2025.729. [Article in French] doi: 10.48327/mtsi.v5i4.2025.729 (PMC12948364; doi:10.48327/mtsi.v5i4.2025.729)
Supplement: Related texts

# Research article: PASS units: mechanisms for delivering healthcare to underserved populations. The example of the Hôtel-Dieu PASS, Paris

## Introduction

The word precarious comes from the Latin *prae-carius,* meaning “poorly secured” and, more broadly, “precarious” meaning fragile and uncertain. Being in a precarious situation means living with constant uncertainty and instability, without any security. Several factors contribute to this situation, including a lack of housing, employment, health insurance, and financial resources. Being sick and living in precarious conditions is a double punishment that complicates medical care and weakens the care pathway. Based on these findings, and in the face of rising poverty rates (16% in 1996) [5], the French public authorities addressed the issue of exclusion. The July 29, 1998 law made fighting exclusion a national imperative based on respect for the equal dignity of all human beings [20].

The law aims to guarantee effective access to fundamental rights throughout the country, including the right to healthcare. The law promotes the creation of medical facilities dedicated to the most disadvantaged, including the uninsured (e.g., certain immigrants in an irregular situation): health care access centers known as PASS (Permanences d’Accès aux Soins de Santé). PASS centers are special to France. A PASS is active in the Hôtel-Dieu (Public hospitals of Paris).

To ensure “effective access for all,” these facilities adapt to the specific constraints and difficulties faced by people in precarious situations. They aim to facilitate access to the healthcare system and provide support in recognizing the rights of the most disadvantaged. At least two activities constitute a PASS: immediate care and access to medium-term health protection.

Recently, the threat of abolishing or limiting state medical aid (AME) under the “Immigration, Integration, Asylum” law of January 26, 2024 has called into question effective access to healthcare for all. AME was not abolished, but the 2025 finance law passed by the Senate nevertheless reduces the AME budget by €111 million compared to the initial proposal [8,21].

This article first presents the organization of PASSs in France and then details the Hôtel-Dieu PASS as an example. The discussion addresses the specific nature and main principles of care for the most vulnerable.

## PASS units in France: 25 years of existence

As of 2023, there were 451 PASSs nationwide. They comply with the instructions and specifications of the Directorate General for Healthcare Provision (DGOS) [10,12].

### Different types of PASS units

The vast majority (380) are general care facilities. There are four types.

- “Dedicated PASS units” offer reception by specific caregivers in a clearly identified, centralized location. These centers have at least one social worker, one nurse, and one doctor. These centers provide comprehensive care through access to general practitioners, technical facilities, nursing care, medication, and specialist consultations. Additional activities, carried out by dedicated staff, may also be provided, such as mobile teams, dental care, and mother/child care. PASS units can either consolidate consultation’s offer or be deployed in hospitals. They are designed to act as a link between hospitals and communities, providing comprehensive medical and social support to people in precarious situations, with the goal of transitioning them back to standard consultations.
- “Cross-disciplinary PASS units” operates throughout the hospital, particularly after a visit to the emergency department, which is often the first point of contact for patients in precarious situations. These consultations are carried out within each department for patients with complex medical and social situations. According to the circular of April 12, 2022, relating to the specifications for PASS units, these teams will gradually disappear because this activity falls within the budget of the Regional Precarity Investment Fund [12].
- “Mobile PASS units” operate “outside the walls”. These teams work closely with vulnerable patients, proactively reaching out to those farthest from the healthcare system in reception and support centers for drug users, reception centers for asylum seekers, camps, and so on. “Health buses” are funded for this purpose. This outreach approach aims to identify vulnerable groups and their medical needs, and refer them to the most suitable medical facilities.
- Finally, there are specialized PASS units, including two in ophthalmology, ten in oral healthcare, and forty-four in psychiatry.

### Creation of City PASS units

Following the successful trial of PASS units in Paris in 2013, City PASS units were created in 2022 as part of the regional program for access to prevention and healthcare. They were designed to support the regional network that provides access to primary care in the community for vulnerable populations.

Thanks to their local roots and network-based approach involving local stakeholders, City PASS units complement the healthcare services offered by hospital PASS units.

Currently, there are 18 operators in the Île-de-France region operating in two ways: in health centers and clinics and through associations formerly part of healthcare networks for people in precarious situations. Ambulatory PASS units facilitate access to healthcare by offering medical and nursing consultations and medication dispensing, made possible through cooperation with local primary health insurance funds (Caisses primaires d’assurance maladie, CPAM). The social aspect is also addressed: a preliminary interview with a social worker identifies vulnerabilities and provides social support in obtaining entitlements and managing the recovery of costs related to healthcare services.

Ambulatory PASS units offer the major advantage of providing local services close to where patients live, facilitating continuity of care, especially after entitlements have been established.

Regional Health Agencies (ARS) finance these initiatives up to €1,390,000. The growing number of active files (a 49% increase from 2020 to 2023) highlights the increasing need for and effectiveness of these structures (Fig. 1) [1].

**Figure 1:** Active patient file recorded for men, women, and children in urban PASS programs in Île-de-France from 2020 to 2023

Source: DGOS report on the 2025 Regional PASS Day

### Target audience

PASS units are intended for anyone in a precarious situation who requires medical care and whose psychosocial situation hinders their integration into the healthcare system [12]:

- Due to non-existent or insufficient social security coverage, making impossible to cover medical expenses.
- For other social reasons, such as difficulty accessing the healthcare system spontaneously, needing support in the care pathway, marginalization, severe social exclusion, significant language barriers, concerns about an irregular legal status in the country, or complex consultations related to mental or psychiatric disorders.

Through its comprehensive support for patients, PASS units act as a bridge, offering temporary support to transition to “traditional” consultations while ensuring continuity of care.

### Intra-and extra-hospital collaborative work

To fulfill their mission of welcoming the most vulnerable people, PASS units must collaborate closely with external partners, such as mobile “health and poverty” teams, “poverty nursing” teams, “psychiatry-precariousness” teams, and outreach teams. They must also collaborate with CPAM (French social security offices), child welfare centers, maternal and child protection centers, local medical-social centers, integrated reception and orientation services, solidarity and integration centers and shelters…

At the same time, the dedicated hospital PASS unit is responsible for raising awareness among hospital professionals about precariousness and identifying people in vulnerable situations. They are induced to refer these individuals to the appropriate structures, particularly PASS units. The 2022 instructions from the DGOS emphasize the importance of coordination between emergency and PASS units. This coordination aims to prevent patients from foregoing care by not going to the emergency room or, conversely, from repeatedly going to the emergency department for non-emergency reasons. The instructions mention the need to establish protocols for identifying and referring vulnerable patients, as well as coordination between physicians, nurses, PASS units, and social workers with the emergency care team.

### Difficulties in accessing care

Despite measures to integrate vulnerable people into the healthcare system, barriers to access to care have been identified.

First, people in extremely vulnerable situations who lack stable housing or reliable access to food tend to neglect medical issues. For these individuals, emergency concerns include access to basic necessities such as food, water for hydration and personal hygiene, and shelter for adequate protection from the weather and safety. Therefore, there is a strong parallel between access to stable housing and access to the healthcare system [17]. This precarious situation creates a different sense of time that dictates the rhythm of homeless people’s lives. The need to focus on daily survival makes it difficult to plan medium-to long-term projects. For people in such precarious situations, planning, organizing, and keeping medical appointments at a specific time and date may seem inappropriate. PASS units usually offer walk-in services to overcome this initial obstacle.

People in precarious situations who are very far removed from the health and social services network do not necessarily have clear knowledge of or a satisfactory understanding of the support structures available to people without financial resources. Due to a lack of awareness that all healthcare costs are covered, these individuals deny themselves access to medical consultations until their situation deteriorates and thus, they must turn to emergency services. In terms of financial issues, traveling to distant medical facilities can be difficult. These economic, social, and geographical constraints further isolate vulnerable populations from healthcare systems.

Limited proficiency in French is a major barrier for migrant populations living in precarious conditions. Without interpretation or language mediation services, these individuals struggle to understand essential medical or administrative information, which limits their access to healthcare. These patients are unaware that PASS units offer these services.

People in precarious situations, particularly undocumented individuals, may be wary of outside help. They may limit their movements for fear of being stopped or checked, especially when going to medical facilities. They may also fear that their medical situation will be exploited for administrative purposes, particularly when the police prefecture issues an Obligation to Leave French Territory (OQTF) or when applying for asylum. In this way, mobile outreach teams and community centers play a crucial role in referring vulnerable people to PASS structures.

### Funding of PASS units

PASS funding comes from the state budget and is managed by CPAMs, ARSs, and hospital administrations.

Before 2022, PASS structures were funded by the Mission d’Intérêt Général (MIG). This program provided PASS units with direct funding via a national grant, which was allocated as part of missions of general interest, as well as assistance with contractual arrangements for healthcare establishments. The MIG credits were financed by a dedicated envelope, distributed by the DGOS, and allocated according to national directives by the ARS, to healthcare establishments.

PASS units received a grant, known as “MIG PASS,” to cover the costs of non-recoverable outpatient care provided within their facilities, as well as management, coordination, and evaluation activities. Hospitalization costs and billable consultations were excluded. This allocation aimed to relieve hospitals of additional staff costs and all costs related to PASS activities, including pharmaceutical expenses, medical examination costs, interpretation services, and transportation costs for mobile PASS units.

Until 2022, the amount of funding allocated to PASS units ranged from a minimum of €50,000 to more than €450,000, depending on the number of people registered.

In 2022, the total funding allocated to the MIG for PASS units amounted to €88,904,676 [11].

In 2022, as part of the Social Security financing bill, these funds were transferred to the Regional Intervention Fund (FIR). This change was intended to simplify and regionalize funding, allowing ARSs to manage these funds directly while reducing the number of MIGs (127 in 2021) and making their objectives clearer. The funds released under the MIG PASS in 2022 were reevaluated and integrated into the FIR in order to benefit from their regionalization. This new organization within the FIR is an attempt to rationalize the budget. Its effectiveness in reducing access inequality will depend on the ARSs’ future ability to adapt their actions to the specific needs of vulnerable populations while preserving the resources necessary for the PASS units to operate smoothly.

The priority highlighted in measure 27 of the 2020 “Ségur de la santé” agreements for the fight against social and territorial health inequalities [13] has made it possible to allocate substantial and long-term funding to the MIG PASS and MIG *Précarité* in an effort to reduce inequalities in access to healthcare. Their consolidation and integration into the FIR aim to deploy a more comprehensive approach to the challenges of precariousness as a whole at the regional level. FIR funding, like MIG funding, is subject to the principle of annual budgetary allocation. However, the ARSs may, either through the multi-year contract on objectives and resources for healthcare establishments or through a specific agreement, provide multi-year visibility to establishments and to professionals.

The amount of FIR funding for 2023 was €105 billion. The ARS allocation for 2023 for the Île-de-France region alone was €30,613,853.

### Activity figures of PASS units

According to data from the monitoring of activity reports on missions of general interest, 206,562 individuals consulted a PASS at least once in 2021, compared with 193,108 in 2018. This figure increased to 252,778 in 2023. Île-de-France accounts for over a fifth of the active patient file (53,330 people).

In 2023, PASS units had 64% new patients in their active patient file (63% in Île-de-France). The average number of medical consultations in Île-de-France was 2.08 per patient, compared to 1.95 in 2022.

The 2023 activity report for hospital PASS units in Île-de-France, compiled by the ARS, reported an average active patient file of 615 patients per medical professional [1].

Social activity in 2023 was 1.71 consultations per patient for an active patient file of 44,801 patients (Fig. 2). In Île-de-France, nursing activity reported 1.52 consultations per patient, for a total of 32,267 consultations in 2023 (Fig. 3).

**Figure 2:** Social activity in Île-de-France in 2023, according to DGOS data, reporting the number of patients on the active patient file of social workers in the PASS units of Île-de-France

Source: DGOS report on the 2025 Regional PASS Day

**Figure 3:** Nursing activity in Île-de-France in 2023 according to DGOS data, reporting the number of patients on the active list of nurses in the PASS units of Île-de-France and number of consultations carried out

Source: DGOS report on the 2025 Regional PASS Day

### Patient profile

The population is predominantly male (57%) and young, with one-third of patients under the age of 25. In 2023, PASS units in France took 8,029 unaccompanied minors (UMs) into care. “Unaccompanied minors” refers to individuals who claim to be minors but have not yet been recognized as such by the state. That same year, the Ministry of Justice officially recognized the minor status of 19,370 of them.

Of the 2,042 UMs registered in Île-de-France, 25% were taken in by PASS units in the country. The proportion of UMs among asylum seekers remains stable at around 4% of all applications submitted.

The UMs’ housing situation is clearly precarious: only 21% have permanent housing, 26% are housed by third parties, and over 50% are in very precarious accommodation. Their lack of health insurance (56%) is another reflection of their vulnerability.

The proportion of the population with an immigrant background is constantly increasing. In 2023, France had 7.3 million regular or irregular immigrants, representing 10.7% of the total population—an increase of 3.8% compared to 2022 and 32% compared to 2010 [6]. Consequently, 74% of individuals receiving PASS assistance are migrants, 30% of whom are from Sub-Saharan Africa (Fig. 4).

**Figure 4:** Geographical origin of PASS' patients in France in 2023.

Source: DGOS report on the 2025 Regional PASS Day

The PASS units have been able to adapt to the needs of immigrant patients, who originally accounted for less than 40% of those receiving assistance.

Over the years, the use of professional interpreters during consultations has increased. In 2023, PASS units across the country provided more than 65,000 additional interpreting services compared to the previous year.

In 2023, the number of non-French speakers in the total PASS active patient file was 58,507, representing approximately one in four patients, compared to 55,641 in 2022. PASS units in Martinique and French Guiana are particularly affected, with 44% and 45% of patients being non-French speakers, respectively. In Île-de-France, 21% of consultations are in a foreign language, totaling 10,205 interpreting services.

Alongside the growing demand for consultations, health coverage reforms implemented on January 1, 2020, introduced measures that complicated access to healthcare for vulnerable foreigners in both regular and irregular situations. These measures include a reduction in the benefits eligibility period, a three-month waiting period for asylum seekers to access the general health insurance system, a three-month presence requirement for all AME applications, and an in-person requirement for submitting initial AME applications at the CPAM (except for specific exemptions granted by hospital PASS units). These changes have restricted access to healthcare for disadvantaged foreign populations, exacerbating their vulnerability and putting more pressure on specific services, such as PASS (see Fig. 5).

**Figure 5:** Change between 2008 and 2022 in the proportion of patients at the Hôtel-Dieu PASS receiving AME, CMU-c/CSS, and not entitled to any benefits.

Source: Internal statistics from the Hotel Dieu PASS

In most cases, care provided by PASS is not billed to patients. A social assessment by social workers may reveal that patients are not eligible for social security coverage at the time of care. Their care is then transferred to the PASS budget. In 2023, 69% of patients seen by PASS units nationwide had no health coverage, totaling 152,805 patients. Ten percent of the 221,457 patients on active waiting lists had AME, and 10% had universal health coverage (PUMA) without supplementary insurance.

The precarious situation of patients received by PASS units is also characterized by their limited financial resources. Seventy-two percent of hospital PASS patients in France have no resources, making access to housing difficult, especially if patients are not eligible for reception conditions under asylum law. According to the DGOS 2023 report, 27% of patients are homeless living on the streets or having occasionally access to emergency accommodations via the social emergency number 115, while 2% live in squats or slums. Additionally, 36% of patients are housed by friends or relatives, and 13% are housed by associations for an extended period. Only 13% report having fixed, stable accommodation.

## The Hôtel-Dieu PASS in Paris: *“Medicus et hospes,* host and doctor”

The Hôtel-Dieu was founded around 660 by Saint Landry, who was an officer in the royal chancellery under the king Clovis II. He received the sick, beggars, and simple pilgrims at his own expense. *“Medicus et hospes,* host and doctor” was the motto of Saint Landry, who was appointed bishop of Paris in 630.

A symbol of hospitality and charity since its creation, the hospital has retained these values, which remain very much alive in its operations today. Its location in the heart of the capital, at the intersection of the regional express network lines, makes Hôtel-Dieu a local hospital for many suburban residents, as well as for those who work or live in Paris.

### Staff and services involved

The Hôtel-Dieu PASS makes it as easy as possible for patients in precarious situations to receive care by organizing multidisciplinary care in one location. The team consists of 10 physicians (equivalent to two full-time positions), including an oncologist, three nurses, three social workers, a social secretary, and a nursing assistant. Hôtel-Dieu participates in training and raising awareness among professionals in the health and social sectors. It does so by welcoming six general medicine residents per semester, four younger nonresident students per quarter, eight young volunteers on a rotating basis throughout the year, four nursing residents, and two social services residents. The young volunteers support patients before and after medical appointments and within various departments.

In addition to the general medical PASS staff, there is also an ophthalmology and dentistry PASS with a volunteer ophthalmologist, three dentists, and three orthoptists on site.

The Hôtel-Dieu radiology department complements the range of care offered by providing a specialized technical platform for PASS patients, who benefit from it in the same way as patients with social security coverage.

The PASS also offers podiatry services through the PODOPASS program. A volunteer podiatrist provides weekly care that would otherwise be difficult to access due to financial constraints. This care is particularly important for vulnerable patients at risk of osteoarticular and infectious complications.

Due to the significant difficulty these patients had accessing eye care, caused by the low number of specialists, the high cost of care in the private sector, and the high price of glasses, an ophthalmological PASS was created in 2016. Thanks to a partnership with One Sight Essilor Luxottica, the PASS has the ability to provide glasses on site. In 2018, a dental PASS was added to the general PASS due to significant patient demand. Patients’ treatment was hindered by the same reasons that affected ophthalmological care. Only prosthetic care is not provided. This issue is currently under consideration.

Recently, we found that physiotherapy has become inaccessible. Even with social security coverage (CSS, formerly CMU-c, or universal supplementary medical coverage) or with AME, many patients cannot receive care. Many say they are refused by practitioners. Others report being asked to pay an additional fee they cannot afford. Since 2021, a partnership with *Rééducateurs Solidaires* has enabled the Hôtel-Dieu PASS to offer, via its “KinéPass” scheme, this essential care on site, as evidenced by the frequency of physical trauma cases.

Since 2022, close collaboration with the psychiatry department and support from the *Fondation de France* have strengthened psychiatric care for vulnerable patients through the implementation of the COMPASS-PSY program. This four-pronged program addresses the high prevalence of mental disorders in this population. First, weekly staff meetings with a psychologist and a psychiatrist allow caregivers to provide care according to the recommendations made, to refer patients appropriately, and to receive training. Secondly, joint doctor/psychologist consultations, known as “companionship” consultations, allow caregivers to develop the knowledge and skills necessary to address psychiatric issues, especially post-traumatic stress disorder, and to provide patients with reassurance and support. Weekly group workshops for patients focused on emotional stabilization complete the program. Finally, dedicated consultations with a psychiatrist are available.

Offering care and holistic treatment within the same healthcare establishment (several PASS units: general PASS, dentistry PASS, ophtalmology PASS, KinePASS, COMPASS-PSY) helps patients understand their care pathway and adhere to treatment. For instance, new patients can undergo systematic screening, especially for infectious diseases, within the same facility thanks to nursing procedures and chest X-rays performed on the same day as the medical consultation. The hospital pharmacy can then dispense treatments. The PASS is supported by associations, particularly the *Amis de la PASS,* which provide human and financial resources to improve access to care for vulnerable patients at Hôtel-Dieu.

### Overall activity

The most disadvantaged patients are welcomed throughout the hospital. However, the presence of a general PASS since 1998 has greatly facilitated their care. In 2023, the PASS provided more than 10,500 medical consultations, 3,200 social interviews, and 4,500 nursing procedures for 4,144 different patients (active cases). Over the past 10 years, the PASS has provided over 100,000 medical and social care services. In 2023, it recorded 75% new patients in its active patient file. That same year, 844 ophthalmological consultations were provided to 691 patients. The dental PASS provided 1,330 consultations to 707 patients in 2023.

### Socio-demographic profile of patients at the Hôtel-Dieu PASS

From 1998 to 2015, the proportion of women consulting the Hôtel-Dieu PASS increased, reflecting the growing proportion of women in migration from sub-Saharan Africa (>40%). Since 2015, however, the trend has reversed, with the proportion of men continuing to grow from 56% to 71%. This percentage differs from the national data and reflects the high number of Asian migrants, primarily Afghan men (Fig. 6).

**Figure 6:** Changes in the male/female ratio among consultants at the Hôtel-Dieu PASS from 2007 to 2023

Source: Internal statistics from the Hotel Dieu PASS

The average age has decreased by nearly 10 years over the last eight years, dropping from 43 to 34. This can be explained by the growing number of consultations dedicated to very young migrants unaccompanied minors (UMs). In 2023, the Hôtel-Dieu PASS conducted 1,714 consultations (over 15% of the active patient file) for UMs, compared to 50 in 2015 (Fig. 7). This situation can be generalized to the rest of France since, according to the mid-2023 Ministry of Justice report on UMs, 15,000 UMs were identified in 2022 compared to 5,000 in 2014 [9].

**Figure 7:** Evolution of the number of consultations dedicated to unaccompanied minors (UMs) between 2017 and 2023 within the Hôtel-Dieu PASS

Source: Internal statistics from the Hotel Dieu PASS

According to a study conducted in 2023 on the assessment of patients’ vulnerability and medico-social conditions, all patients have one or more vulnerability factors [14] :

- A quarter of patients seen in consultations sleep on the streets; 32% are housed by a third party; 32% are taken in by an association; and less than 10% have their own home (Fig. 8).

**Figure 8:** Distribution of different types of residence reported by patients registered with the Hôtel-Dieu PASS in 2023

Source: 2023 point-in-time survey involving 432 third-party assessments
- Nearly two-thirds do not have access to three meals a day for financial reasons (Fig. 9).

**Figure 9:** Difficulty accessing meals due to lack of resources among Hôtel-Dieu PASS patients in 2023

Source: 2023 point-in-time survey involving 432 third-party assessments
- Half have no health insurance; 17% have access to AME; 24% to CSS (social health insurance for low-income individuals); and 10% have access only to Social Security, without supplementary insurance (Fig. 5).
- Nearly half of the patients receive social services outside the unit, and 60% are referred by a partner association.

In 2023, more than 100 countries of origin were represented, reflecting an increasing trend in diversity. For example, 2% of patients were from France in 2023, compared to 13% in 2015. The proportion of patients from sub-Saharan Africa remains high (61% in 2023 *versus* 53% in 2015), while the proportion of patients of Asian origin is growing (21% *versus* 11%). Those from the Maghreb and Europe remain stable (6% and 3%, respectively, *versus* 8% and 7% in 2015) (Fig. 10). Regarding the languages used by patients, the statistics are consistent with those observed in the Île-de-France region [16]. In 2023, 21% of consultations were conducted in a foreign language. The most commonly spoken languages were Pashto (spoken in Afghanistan and Pakistan), Dari (spoken in Afghanistan), and Bengali (spoken in Bangladesh and India). For 60% of consultations, the communication was considered difficult by the doctor (Fig. 11), which partly explains the increase in requests for professional interpreters: There were 1,244 calls, accounting for 21% of consultations, compared with 4% in 2018 (Fig. 12).

**Figure 10:** Distribution by geographical origin of patients in the Hôtel-Dieu PASS at the between 2008 and 2022

Source: Internal statistics from the Hotel Dieu PASS

**Figure 11:** Assessment of communication difficulties experienced by physicians during consultations with patients at the Hôtel-Dieu PASS and changes between 2018 and 2023

Source: Internal statistics from the Hotel Dieu PASS

**Figure 12:** Changes in the use of professional interpreters between 2008 and 2023

Source: Internal statistics from the Hotel Dieu PASS

### Pathologies observed

The reasons for consultation are diverse. However, some general trends are emerging. Reasons related to trauma and infectious diseases are overrepresented. Reasons related to psychiatry and gastroenterology are more common than in the general population (1 in 4 patients). Conversely, consultations for viral infections are very low (5%) (Fig. 13).

**Figure 13:** Diagnosis/Diagnoses established at the end of the consultation by the PASS doctor at Hôtel-Dieu

Source: 2023 point-in-time survey involving 432 third-party assessments

The most common conditions leading to consultations in 2023 were as follows:

- High blood pressure (HBP). The HBP rate is 19.2%. HBP particularly affects people over 40 (44%). It is often diagnosed at severe levels (>180/110 mmHg). As a reminder, nearly one in three adults in mainland France has high blood pressure, especially in older age groups. The difference between the general population and the PASS population is that the latter is affected by HBP at an earlier age and more severely.
- Diabetes. Diabetes accounts for 12.6% of consultations, with 10% being non-insulin-dependent and 2.6% being insulin-dependent. It affects 29.9% of people over the age of 40, or nearly one in three patients [14]. As a reminder, the prevalence of diabetes in France is 5.6% [18]. Patients have higher HbA1c levels than the national average, less regular follow-up and more frequent micro-angiopathies [7].
- Viral hepatitis B. Hepatitis B is particularly prevalent, with an incidence rate of 9.9% in 2022 (95% CI: 7.5-12.3). For reference, the prevalence in mainland France in 2016 was estimated at 0.3% (0.13-0.70) [15] (Fig. 14).
- Viral hepatitis C. The prevalence of active hepatitis C (defined by positive serology and viral load) is 1.14% among PASS patients, which is four times higher than the prevalence in the general adult population of metropolitan France. In 2016, the Health Barometer estimated the prevalence of active hepatitis C at 0.3% (95% CI: 0.13-0.70) [15] (Fig. 14).

**Figure 14:** Estimated annual number of HBsAg-positive cases, active hepatitis C cases (missing data 2020), and HIV cases (missing data 2020) within the Hôtel-Dieu PASS.

Source: Hotel Dieu PASS Steering Committee 2023
- HIV infection. The HIV infection rate is estimated at 1.17% in 2022. This data should be monitored over the next few years. According to *Santé publique France,* the number of new HIV diagnoses is increasing between 2021 and 2023, particularly among individuals born outside of France [16], as shown in Fig. 15.

**Figure 15:** Trends in newly diagnosed HIV cases in France by population group (defined by gender, probable mode of transmission, and country of birth), from 2012 to 2023 MSM: Men who have sex with men

Source: Mandatory HIV Reporting (DO VIH), Data as of 06/30/2024, adjusted for reporting delays, underreporting, and missing values
- Tuberculosis. Around 30 cases of tuberculosis are diagnosed each year among the 4,000 patients seen. According with the French Society of Infectious Diseases, the incidence rate among people born outside of France (31/100 000) is approximately 10 times higher than among those born in France. The disease particularly affects the poorest sections of the population, especially homeless people, among whom the incidence rate (around 61 per 100,000 people) far exceeds that of other social groups [19].
- Latent tuberculosis infections. In 2022, 300 interferon gamma release tests were performed within the PASS among minors and migrants from high-prevalence areas to identify latent tuberculosis cases. Thirty-five percent of the tests were positive. For comparison, 23% of the world’s population had latent tuberculosis in 2014. Screening and treating latent tuberculosis infections is a major public health challenge given the risk of an increase in emerging tuberculosis cases transmitted by migrants, especially in countries with low tuberculosis incidence [4].

### Vaccinations

In 2022, 2,700 vaccinations were administered at the Hôtel-Dieu PASS to bring people up to date on their vaccinations according to the French recommendation schedule. The main vaccines administered were:

- the conjugate vaccine against diphtheria, tetanus, polio, and pertussis;
- the measles, mumps, and rubella vaccine;
- the hepatitis B vaccine.

Other vaccinations carried out at the PASS included the meningitis ACWY vaccine (for individuals under 25), the pneumococcal vaccine (as indicated), the hepatitis A vaccine (mainly for patients with HBV infection), and the SARS-CoV-2 vaccine. Vaccination against HPV (Human Papilloma Virus) is not yet offered, for cost reasons.

### Psychological and psychiatric care

This area of care is growing as many patients suffer from mental distress or post-traumatic stress disorder related to their past or migration experience. Their care is multidisciplinary and complex and requires close monitoring. Twenty-two percent of people who have experienced war or other conflict situations in the last 10 years suffer from depression, anxiety, post-traumatic stress disorder, bipolar disorder, or schizophrenia [2]. Healthcare staff are trained to treat these conditions. This appropriate care makes the PASS a preferred place of refuge for people experiencing extreme psychological and psychiatric vulnerability.

### Discussion

Since PASS operations vary greatly from one facility to another, it is difficult to compare their activities. However, the Hôtel-Dieu PASS allows us to identify constraints common to all PASS units. Since its creation, the activity of the Hôtel-Dieu PASS has grown steadily, as has that of other PASS units in the country. The patient profile and geographical origin have evolved over the years in line with geopolitical conflicts. Currently, a large proportion of the population is from immigrant backgrounds, and they often require urgent and regular medical follow-up due to their state of health.

Caring for sick people and those in socially vulnerable situations is complex. The actions taken are lengthy and time-consuming, and they only become effective over the long term.

The complexity of this care could create social disparities in access for these patients and lead healthcare professionals without the necessary training, resources, or structure to become resigned or indifferent. PASS units’ mission is to reach these highly vulnerable patients, identify obstacles to their care, reintegrate them into the system, and then refer them to non-specific structures.

These individuals reveal social dynamics and structural dysfunctions that must be analyzed and understood. Although technical care is indispensable and undeniably beneficial, the care of human beings in a healthcare setting cannot be limited to this dimension alone. A holistic medical approach highlights the vital importance of interpersonal relationships, which play a complementary and essential role in the healing process. This approach considers individuals holistically, integrating their physical, emotional, mental, social, and spiritual dimensions to promote harmonious, lasting balance. PASS units, including the one at Hôtel-Dieu, are particularly affected by changes in society. According to Xavier Emmanuelli, “The world is in motion, and we cannot stop it.” We will not engage in political debates about migration here. Instead, we will reiterate the Hippocratic Oath, which is a red line that needs to be emphasized today: “I will respect all persons, their autonomy, and their will without discrimination. I will intervene to protect them if they are vulnerable or threatened in their integrity or dignity…”. When faced with a patient, physicians are not there to judge the merits of migration policies. They are faced with a suffering person who must be relieved by all possible means.

After 25 years, PASS units have proven their value in the healthcare system. They treat patients with complex social and medical situations, offering appropriate, individualized, and comprehensive medical and social care. PASS units are an alternative to emergency department visits and medical “zapping” for this population. PASS units reflect the evolution of French society and must adapt to changing demands, which fluctuate with world evolution. An example of this is their role in helping unaccompanied foreign minors who are left to fend for themselves in our country.

PASS facilities are an underutilized observatory of the health of a marginalized segment of the population. Above all, they are and must remain a place of care.

These facilities require strong financial support from the state, local support from regional health agencies and hospital management, and appropriate professional commitment. They are inherently fragile and unprofitable in the short term. They are also wondering by the population they serve and are disruptive due to the specific organization they require.

### Specificity of care for the most vulnerable

With 25 years of experience working with the most disadvantaged, the Hôtel-Dieu PASS has identified several findings that should be considered when establishing the main principles for caring for vulnerable people.

- The more destitute the patients are, the more important it is to consider the social context when designing the care plan and selecting the treatment. Social precariousness takes precedence over the medical dimension and must be considered first.
- The more socially vulnerable and culturally different the patients are, the more we need to adapt our system and the way we welcome them to facilitate their access to care.
- The loss of spatial and temporal reference points among homeless people is key to understanding “missed appointments.” We must draw conclusions from this phenomenon, as unity of place and time of care are essential prerequisites. Walk-in services further enhance the facility’s ability to adapt to the schedules of people in extremely precarious situations.

The role of PASS is to develop means of providing optimal care for people in precarious situations. PASS strives for the best possible outcome, but does not fixate on it, as “success” is rare.

### Key principles of medical care for vulnerable populations

#### Identify vulnerability factors

The main vulnerability factors to identify are as follows:

- **Housing:** In 2023, 15 million people in France were considered to be in fragile housing situations, including 4.1 million who were “unhoused or poorly housed” [3]. During the interview, it is useful to find out which neighborhood the patient lives in, whether they sleep on the street, how many people live in the same room, if they receive any housing assistance, and if so, in what form (financial, household chores, or sexual favors). It is also useful to find out if they call “115,” (phone number for social assistance), etc.
- **Precarious housing** can lead to health issues such as carbon monoxide and lead poisoning, chronic bronchitis, asthma, tuberculosis, osteoarthritis, anxiety and depression, headaches, eczema, bacterial, parasitic, and fungal skin infections, and sleep disorders. Better identification of living conditions makes it possible to optimize screening and tailor treatment to the patient.
- **Job insecurity** leads to financial insecurity, housing difficulties, a breakdown of social ties (including with family members who remain in the country of origin), a lack of routine, low self-esteem, and reduced social recognition.
- **Food insecurity**, in terms of quantity, can cause weight loss; in terms of quality, it often leads to being overweight and deficiencies in iron, folate, vitamin B12, vitamin C, and protein.
- **Financial poverty** is assessed by precarious housing and employment and difficulty accessing food.
- **Isolation** is often exacerbated by language barriers and substance abuse.
- **Precariousness linked to migration:** while “being a migrant” is not equivalent to “being in a precarious situation,” special attention must be paid to migrants, as many currently live in difficult conditions. They may be in an irregular administrative situation, unable to work legally, and have limited financial resources. Language and cultural barriers further isolate them and impede their understanding of the healthcare system and constantly changing immigration regulations. The fear of deportation associated with their irregular status makes them distrustful of administrative and medical institutions, which encourages them to avoid healthcare services. The lack of adequate housing exacerbates their psychological suffering related to exile, their migration journey, lack of integration, and violence they have experienced.

#### Listen to suffering linked to precariousness

Listening is an essential step in the therapeutic process, especially for vulnerable or precarious populations. It enables individuals to express themselves, a practice that is often overlooked, and fosters a relationship between caregivers and patients, which is crucial for providing appropriate care. This process can be compared to comfort care, where the goal is to improve quality of life by addressing the patient’s immediate needs. In both cases, listening plays a central role in responding to urgent and exclusionary situations.

In a context of temporality and immediacy where patients’ expectations of the healthcare system are often urgent and follow-up is inconsistent, listening enables one to transcend mere functional exchanges and foster mutual understanding. This helps patients plan ahead and strengthens their commitment to care. For people in precarious situations, this approach combats social and medical exclusion by addressing their specific issues in their overall care. It offers solutions tailored to their living conditions and optimal treatments adapted to their psychosocial context. Additionally, this active listening approach is part of a “reach-out” strategy, which is particularly relevant for people distant from the healthcare system, as it creates a link and initiates medical or social follow-up. Thus, listening becomes a powerful tool for establishing a therapeutic relationship and reintegrating vulnerable populations into the healthcare system.

#### Offer multidisciplinary medical and social care as part of a network

The medical and social complexity of patients in precarious situations requires a collaborative, multidisciplinary approach. Partnering with social teams and community partners is essential for providing effective care to these patients. These patients often present complex problems involving physical health, mental disorders, social vulnerability, and administrative difficulties, which can only be resolved through close collaboration between social workers, healthcare teams, and community partners.

Social workers play a pivotal role in finding practical solutions for patients, including access to medical care, housing, food and clothing assistance, and legal support. Their intervention enables patients to meet their basic needs, which are often prerequisites for their adherence to medical care. Caritative associations provide complementary expertise and logistical support for targeted actions, such as emergency accommodation, psychological support, and cultural mediation. They significantly reduce barriers to healthcare by offering services tailored to vulnerable groups. They often identify patients’ unspoken medical needs and organize their first appointment with the PASS.

This cross-disciplinary collaboration aims to break down the barriers between medical and social services to effectively respond to vulnerable patients’ complex needs and embed itself in measure 27 of the “Ségur healthcare agreements” to reduce access inequality. The PASS system fully demonstrates its role as a bridge by reintegrating these patients into society and empowering them in the long term.

#### Support, then empower

Despite efforts to simplify and digitize them, the healthcare system and immigration procedures are still difficult to understand and navigate. At the start of their treatment, patients are very often dependent. Optimal care requires scheduling medical appointments and providing physical assistance to reach radiology departments, pharmacies, and so on.

Organizing a colposcopy, for example, illustrates these difficulties: Patients must attend several appointments with the specialist so they can make an indication of examination and explain the procedure, ideally in the patient’s native language or through translation. The consultation must detail not only the procedure itself but also how to prepare for it.

Organizationally, it is necessary to print a map of the hospital where the procedure will take place (which is sometimes different from the consultation location). It is also necessary to ensure that the patient understands by asking them to repeat the directions.

These difficulties are compounded by language barriers or illiteracy.

The empowerment stage comes later and is gradual, fraught with many “setbacks.” The caregiver must persevere, not feel guilty, and find ways to work with the patient.

#### Promote unity of place and time in care

People in highly precarious and vulnerable situations often have limited ability to plan for the future because their daily lives are consumed by the urgent search for housing and food.

This context fosters an immediate approach, where needs must be met “here and now.” This prioritization of urgency makes approaches requiring long-term vision difficult.

Therefore, whenever possible, long-term follow-up is offered to help these patients develop a vision for the future. Providing continuity of care creates a stable environment, which is essential for promoting personal recovery. Having a fixed and regular place and time for care provides patients with a strong framework on which they can gradually rebuild their lives and move beyond the logic of immediate survival.

#### Adapt treatment and advice to living conditions

The number, timing, and methods of treatment should be tailored to living conditions, particularly for individuals experiencing homelessness. Patients’ difficulty in accessing the treatment center must be assessed.

The choice of medication and treatment cannot be made without prior knowledge of the patient’s living conditions and constraints.

## Conclusion

Caring for people who need medical care and face social difficulties is a complex, ongoing challenge where medical and social vulnerabilities intersect, limiting the effectiveness of individual interventions. Many problems persist, such as financing additional tests, medication, and hospitalizations, as well as integrating patients into the healthcare system. PASS units must rise to the challenge of caring for the most vulnerable people, who are the farthest from the healthcare system, by promoting their potential. A lack of care would have serious consequences for these individuals and the community in terms of health (e.g., infectious risks) and finance (e.g., cost of untreated complications). Supporting vulnerable, highly precarious, migrant, and marginalized populations is not just an option for French society; it is an ethical and humanitarian imperative.

## Acknowledgments

We would like to express our sincere gratitude to Prof. Eric Caumes for his support and advice for our patients, and to Prof. Eric Pichard for his insightful comments.

## Funding

No industrial or commercial funding was received for this work.

## Conflicts of interest

No conflicts of interest have been declared.
